# CD4^+^ Effective Memory T Cell Markers GBP2 and LAG3 Are Risk Factors for PTB and COVID-19 Infection: A Study Integrating Single-Cell Expression Quantitative Trait Locus and Mendelian Randomization Analyses

**DOI:** 10.3390/ijms25189971

**Published:** 2024-09-16

**Authors:** Liangyu Zhu, Hanxin Wu, Li Peng, Xun Huang, Rui Yang, Weijie Ma, Lei Zhong, Bingxue Li, Jieqin Song, Suyi Luo, Li Gao, Xinya Wu, Weijiang Ma, Fukai Bao, Aihua Liu

**Affiliations:** 1 Yunnan Province Key Laboratory of Children’s Major Diseases Research, Department of Pathogen Biology and Immunology, School of Basic Medicine, Kunming Medical University, Kunming 650500, China; 20231847@kmmu.edu.cn (L.Z.); 20221746@kmmu.edu.cn (H.W.); pengli@kmmu.edu.cn (L.P.); 20230028@kmmu.edu.cn (X.H.); 20230029@kmmu.edu.cn (R.Y.); 20220023@kmmu.edu.cn (W.M.); 20220022@kmmu.edu.cn (L.Z.); libingxue1@kmmu.edu.cn (B.L.); 20211707@kmmu.edu.cn (J.S.); luosuyi@kmmu.edu.cn (S.L.); 20210039@kmmu.edn.cn (L.G.); 20210037@kmmu.edu.cn (X.W.); 20221745@kmmu.edu.cn (W.M.); 2 Yunnan Provincial Key Laboratory of Public Health and Biosafety, School of Public Health, Kunming Medical University, Kunming 650500, China

**Keywords:** pulmonary tuberculosis, COVID-19, scRNA-seq, eQTL, T cell subsets

## Abstract

Observational studies indicate that variations in peripheral blood mononuclear cell (PBMC) subsets are associated with an increased risk of pulmonary tuberculosis (PTB) and coronavirus disease 2019 (COVID-19), but causal validation is lacking. Here, we combined single-cell expression quantitative trait locus (sc-eQTL) and two-sample mendelian randomization (MR) analyses to elucidate the causal relationship between PBMC subsets and the occurrence of PTB and COVID-19 and verified by RT-qPCR. We observed an increase in the CD4^+^ Effective Memory T Cell (CD4^+^ T_EM_) cluster in both PTB and COVID-19 patients according to the single-cell transcriptional landscape of PBMC. Through MR analysis using an inverse variance weighted (IVW) method, we found strong evidence of positive correlations between CD4^+^ T_EM_ cell markers (GBP2, TRAV1-2, and ODF2L) and PTB, and between markers (LAG3 and SLFN5) and COVID-19, especially highlighted by lead eQTL-SNPs of GBP2 (rs2256752, *p* = 4.76321 × 10^−15^) and LAG3 (rs67706382, *p* = 6.16× 10^−16^). Similar results were observed in validation sets, and no pleiotropy was detected in sensitivity analyses including weighted median (WM), MR-Egger, MR-pleiotropy residual sum and outlier, and leave-one-out analyses (all *p* > 0.05). We visualized the colocalization of marker-eQTLs and markers of PTB and COVID-19 genome-wide association study (GWAS) associations. Based on CellChat analyses, monocytes communicated predominantly with CD4^+^ T_EM_ cells positively expressing PTB markers (GBP2, TRAV1-2, and ODF2L) and COVID-19 markers (LAG3 and SLFN5) in both PTB and COVID-19. Our data suggest a causal effect between two key CD4^+^ T_EM_ cell markers (GBP2 and LAG3) and the risk for PTB and COVID-19 infection. Our findings provide novel insights into the biological mechanism for PTB and COVID-19 infection, but future single-cell studies are necessary to further enhance understanding of this find.

## 1. Introduction

*Mycobacterium tuberculosis* (*MTB*) causes pulmonary tuberculosis (PTB), a chronic respiratory infectious illness that threatens public health [1]. The World Health Organization’s 2022 Global TB Report indicated that if left uncontrolled, tuberculosis (TB) will become the second leading cause of death from a single infectious disease after coronavirus disease 2019 (COVID-19) [2]. Similar to PTB, COVID-19 is a respiratory-transmitted viral infection involving a novel coronavirus strain, SARS-CoV-2, which primarily infects the lungs. The COVID-19 pandemic has unraveled years of global advancements in the fight against TB, leading to a resurgence in TB-related mortality. Notably, for the first time in more than a decade, deaths due to PTB have risen [2]. A multicenter study showed that PTB/COVID-19 co-infection occurs in 1.5% of COVID-19 patients [3]. Moreover, the incidence of PTB in SARS-CoV-2-infected patients is three times higher than that of healthy individuals [2]. Coinfection with PTB and COVID-19 leads to a delayed immune response to SARS-CoV-2, leading to exacerbation of the disease [4]. A study has demonstrated a high rate of missed diagnoses in patients with COVID-19 and concurrent conditions [5]. Therefore, identifying characteristics common to patients with PTB and COVID-19 is essential for developing strategies to treat the coinfection.

An imbalance in immune cell status is responsible for the occurrence of PTB/COVID-19 coinfection. COVID-19 induces an excessive cellular immune response, leading to functional exhaustion and T-cell depletion [6]. There is direct evidence that patients coinfected with PTB and COVID-19 have a reduced in vitro response to SARS-CoV-2, while their *MTB*-specific response remains unimpaired [7]. Pathologically, SARS-CoV-2 disrupts the balance of granuloma formation, which normally results from proper immune cell regulation [5,8]. Conversely, the PTB-associated chronic impairment of pulmonary and local immunity makes the body more susceptible to acute infections with airborne pathogens [9]. *MTB* affects immune cell function in many ways that delay the immune response and viral clearance, further exacerbating the disease and increasing the risk of disease transmission. The effects of *MTB* include inhibition of apoptosis [10], prevention of antigen presentation by dendritic cells [10], and induction of TB-specific regulatory T-cell expansion [11]. Both pathogens contribute to an imbalanced immune-inflammatory response, leading to disease progression and deterioration.

Mendelian randomization (MR) is a novel approach applied in genetic epidemiology because it is less susceptible to interference from confounders and reverse causation [12]. Noncoding genetic variants can contribute to disease progression by influencing gene expression, and immune cell-based gene expression quantitative trait locus (eQTL) analyses have been widely used, particularly in elucidating the pathogenesis of TB [13]. Recent studies with eQTL analysis missed precision due to the use of bulk tissue data, which ignores the regional, temporal, and cell-type specificity of gene expression regulation [14,15]. Thus, single-cell sequencing, rather than bulk gene expression datasets should enable an assessment of the impact of genetic variants on cell types and activation between PTB and COVID-19.

This study aimed to explore the impact of genetic variants on marker expression of PTB and COVID-19 various PBMC subsets through eQTL mapping at the single-cell level. Notably, we demonstrated for the first time the association of PTB and COVID-19 at the single-cell level and identified a causal effect for markers of CD4^+^ effective memory T cell (CD4^+^ T_EM_) cells on PTB and COVID-19.

## 2. Results

**Figure 1:**
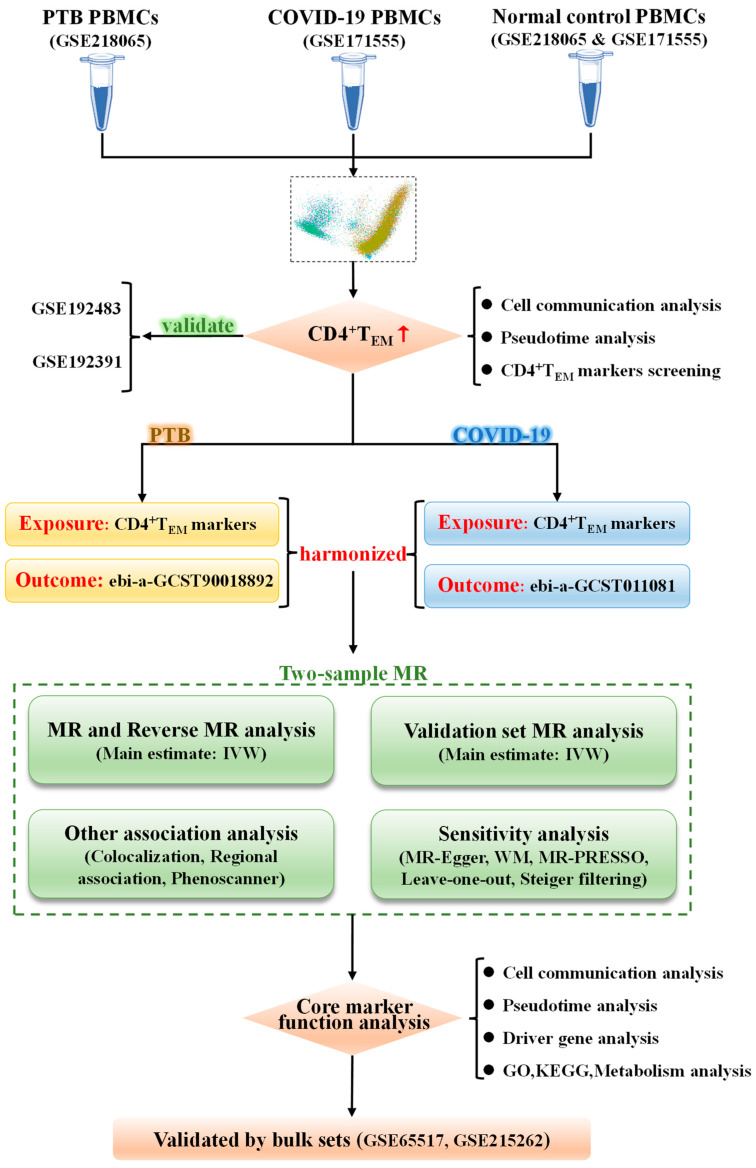
Flow chart of this MR. PBMC = peripheral blood mononuclear cell. PTB = pulmonary tuberculosis. COVID-19 = coronavirus disease 2019. MR = mendelian randomization. IVW = inverse variance weighted. WM = weighted median. GO = gene ontology. KEGG = kyoto encyclopedia of genes and genomes. Notes: The red arrow represents activated cell subsets in both PTB and COVID-19 patients and the black arrows represent the analysis process.

The workflow is visualized in Figure 1. The sources of all single-cell sequencing and genome-wide association study (GWAS) data sources used in the present research are listed in Table 1.

### 2.1. Single-Cell Transcriptional Landscape of PBMCs from PTB and COVID-19 Patients

**Figure 2:**
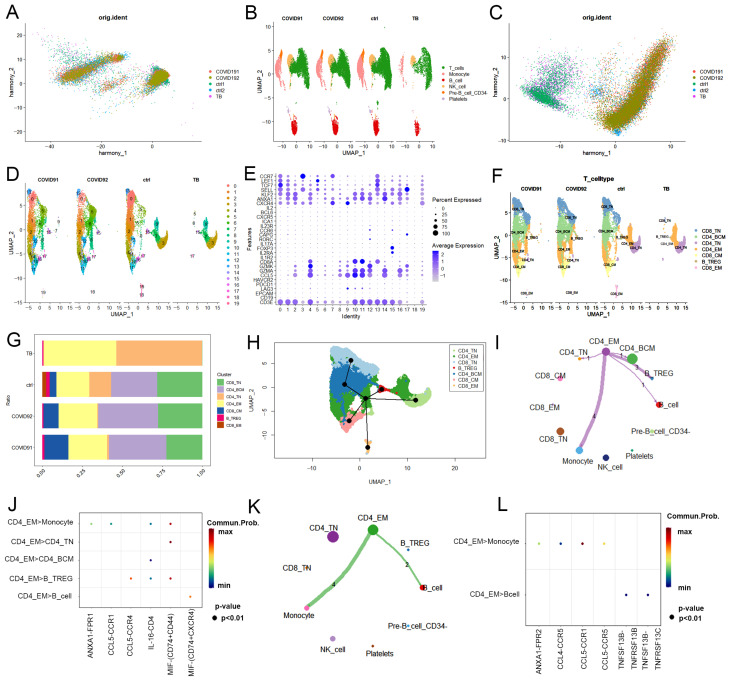
Single-cell transcriptional landscape of PBMCs from PTB and COVID-19 patients. (**A**) Degree of PBMC correlation fitting between PTB and COVID-19 patients after removal of the batch effect as reflected by the principal components analysis (PCA) scatter plot. (**B**) The spatial distribution of each cell subset is named and visualized using uniform manifold approximation and projection (UMAP). (**C**) Degree of T cell correlation fitting between PTB and COVID-19 patients after removal of batch effects as reflected by PCA scatter plot. (**D**) Spatial distribution of each T cell subset named and visualized using UMAP. (**E**) The average expression levels of classical marker genes for every T cell subset. Dot size indicates the percentage expression of selected cellular markers in each subset, and dot color and shade indicate the marker’s mean expression level. (**F**) Visualization of T cell subsets by UMAP diagram and (**G**) histogram. A single cell colored by cell cluster information is represented by each point. (**H**) Single-cell gene expression in a seven-dimensional PCA plot with branching lineage trajectories determined with the “Slingshot” package of R. (**I**–**L**) Network of cell-cell communication that shows the quantity of receptor-ligand pairings between CD4^+^ T_EM_ cells and other T cell subsets associated with PTB (**I**,**J**) and COVID-19 (**K**,**L**). The line thickness indicates the number of pairs. The number of significant ligand-receptor pairs between any two cell populations. Note: CD8^+^ T_N_ (CD8^+^ naïve T cells), CD4^+^ T_BCM_ (CD4^+^ blood central memory T cells), CD4^+^ T_N_ (CD4 naïve T cells), CD4^+^ T_EM_ (CD4^+^ effector memory T cells), CD8^+^ T_CM_ (CD8^+^ central memory T cells), CD4^+^ T_BREG_ (CD4^+^ blood regulatory T cells), and CD8^+^ T_EM_ (CD8^+^ effector memory T cells).

To characterize the cell types and gene expression profiles of PBMCs in PTB and COVID-19, PBMCs isolated from GSE218065, which included 1 PTB patient (TB) and 1 control (ctrl-1), and GSE171555, which included 2 COVID-19 patients (COVID-19-1 and COVID-19-2) and 1 control (ctrl-2), were analyzed. COVID-19 patients were confirmed by positive SARS-CoV-2 PCR and/or immunoglobulin G (IgG) seroconversion. Specific data regarding each patient is provided in Table 1. After filtering out low-quality cells (criteria: minGene = 200, maxGene = 5000, pctMT = 20), transcriptome files for 46,022 cells—an average of 2000 cells per person in each sample—were obtained (Figure S1). Harmony was used to integrate data from PTB and COVID-19 cells and evenly mix the batches (Figure 2A). Principal component analysis (PCA) was performed for initial dimensionality reduction, selecting 10 principal components with high scores (Figure S2). Clusters were visualized using a two-dimensional uniform manifold approximation and projection (UMAP) dimensionality reduction algorithm (Figure S3). Figure 2B shows six major cell clusters with clear borders, including T cells (the most represented), monocytes, B cells, NK cells, pre-B CD34^−^ cells, and platelets (Figures S4–S6). We then integrated cells from the two control samples and compared them between groups to identify T cell subsets in which PTB resembled COVID-19 (Figure 2C,D). It is generally recognized that T cells primarily mediate the immune response against *MTB* and SARS-CoV-2 [16]. Therefore, seven subsets of T cells were clustered, including CD8^+^ naïve (CD8^+^ T_N_), CD4^+^ blood central memory (CD4^+^ T_BCM_), CD4 naïve (CD4^+^ T_N_), CD4^+^ T_EM_, CD8^+^ central memory (CD8^+^ T_CM_), CD4^+^ blood regulatory (CD4^+^ T_BREG_), and CD8^+^ effector memory (CD8^+^ T_EM_) T cells (Figure 2E,F). CD4^+^ T_EM_ cells exhibited a notable increase in both the PTB and COVID-19 cohorts compared to the control group (Figure 2G) and validated by PTB lung tissue and COVID-19 PBMC (Figures S7 and S8). Through pseudotime analysis, these T cells, characterized by shared patterns of gene expression, clustered together and delineated a relative cellular trajectory across simulated time. The cell pseudotime analysis showed that CD8^+^ T_N_ cells and CD4^+^ T_BCM_ cells are activated in the early stages of T cell development, whereas CD4^+^ T_N_ cells represent the terminal differentiation stage (Figure 2H). Notably, CD4^+^ T_EM_ cells track throughout the development of peripheral blood T cells both in PTB and COVID-19 patients (Figure 2H). To further ascertain whether the function of CD4^+^ T_EM_ cells is similar in the two infections, we performed CellChat analysis based on ligand-receptor interactions. The results showed that CD4^+^ T_EM_ cells interact with monocytes and B cells primarily in the peripheral blood of both PTB and COVID-19 patients (Figure 2I–L).

Together, these results demonstrated that increased activation of CD4^+^ T_EM_ cells was considerably strong in patients with *MTB*/SARS-CoV-2 infection.

### 2.2. MR Analysis of CD4^+^ T_EM_ Cluster Markers and PTB

**Figure 3:**
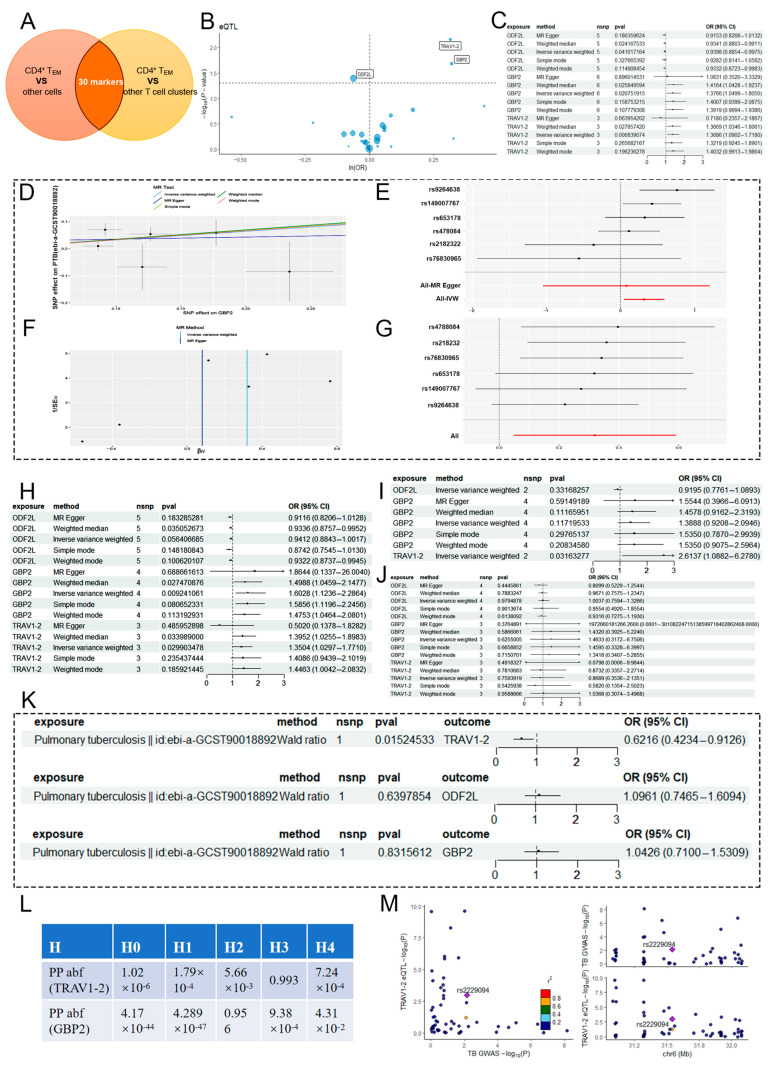
MR analysis of CD4^+^ T_EM_ cluster markers and PTB. (**A**) Venn diagram showing the screening strategy for CD4^+^ T_EM_ cluster markers. (**B**) Volcano plot of the MR results for three markers and the risk of PTB determined using the IVW method. Ors for increased risk of PTB are expressed as per standard deviation increase in marker level. (**C**) Forest plot of five MR model results for the three markers. (**D**) Scatter plots of the five MR models. Each point represents an IV, the line on each point represents the 95% CI, the ordinate shows the effect of the SNP on the outcome, and the abscissa shows the effect of the SNP on exposure. (**E**) Forest plot of MR analysis results for single SNP estimation of GBP2. The red line represents the pooled results for all SNPs (**F**) Funnel plot of three SNPs on MR analysis. (**G**) MR sensitivity results for GBP2 after removing SNPs using the leave-one-out method. The red line represents the pooled results for all SNPs (**H**–**J**) Forest plot of MR validation through ebi-a-GCST90018672 (**H**), bbj-a-149 (**I**), and finn-b-TBC_RESP (**J**). (**K**) Forest plot of reverse MR (R-MR) analysis. (**L**) Results of colocalization analyses, with posterior probability. (**M**) Regional association plot of GWAS results and marker-eQTLs at the marker, PTB, and PTB locus. SNPs are colored based on LD (*r*^2^) with the lead marker-eQTL (rs2256752). Purple diamonds represent the lowest *p*-value for each locus.

To understand the genetic relationship between CD4^+^ T_EM_ cells and PTB, markers of CD4^+^ T_EM_ cells were selected and analyzed using MR through a GWAS. First, 30 key markers of the CD4^+^ T_EM_ cluster were screened by taking the intersection of two different genomes (Figure 3A, Table S1). Gene ontology (GO) analysis of these key markers identified significant immune-related GO terms, including “regulation of innate immune response” (BP), “T cell receptor complex” (CC), and “MHC protein binding” (MF, Figure S9A). Kyoto encyclopedia of genes and genomes (KEGG) analysis indicated that the terms of CD4^+^ T_EM_ markers were related to the occurrence of COVID-19 (Figure S9B).

eQTL mapping is a powerful method for studying how common genetic variations among individuals affect gene expression [17]. To investigate the role these markers, play in PTB, 97 eQTLs were found to be connected to the expression of 30 markers after clumping SNPs that were in linkage disequilibrium (*r*^2^ < 0.001). The mean *F* statistic for the SNPs used as instruments ranged from 29.9 to 2863.3, indicating strong instrumental variables (Table S2). Public GWAS data were used for a two-sample MR study with eQTL SNPs as instrumental variables, markers as exposure variables, and PTB as the outcome variable. The MR analysis suggested that a total of 3 CD4^+^ T_EM_ markers had a direct causal relationship with the development of PTB. Among these, guanylate-binding proteins 2 (GBP2) (IVW, 6 SNPs, *p* = 0.02) and TRAV1-2 (IVW, 3 SNPs, *p* = 0.006) are risk factors for PTB, whereas ODF2L (IVW, 5 SNPs, *p* = 0.04) is a protective factor (Figure 3B,C, Table 2). We also produced scatter (Figure 3D), forest (Figure 3E), funnel (Figure 3F), and leave-one-out (Figure 3G) plots of GBP2 for further interpretation.

For MR validation, the PTB validation sets from patients of different ethnicities confirmed the reliability of the 3 markers (Figure 3H–J). No evidence for heterogeneity (all *p* > 0.1, Table 3), pleiotropy (all *p* < 0.05, Table 3), or reverse causality (all *p* > 0.05, Figure 3K) was observed. The MR reliability of GBP2 was validated by scatter (Figure S10A), forest (Figure S10B), funnel (Figure S10C), and leave-one-out (Figure S10D) plots. Among all SNPs for GBP2, the greatest risk factor for PTB, rs653178, had the maximum F statistic and most significant *p*-value for correlation with GBP2 (*F* = 223.35 and *p* = 1.6638 × 10^−50^). Near 1,000,000 bp of this lead SNP, the genetic associations of all SNP loci with two phenotypes (GBP2 eQTL and PTB) were tested. The result showed that these SNPs had a 4.31% probability of being associated with both phenotypes, implying a low probability of colocation (PP abf of H4 = 4.31%, Figure 3L). In addition, regional association plots better defined the distribution of SNPs near the lead SNP and the synergy with these two phenotypes. Compared to GBP2, TRAV1-2, and PTB have a greater probability of sharing genetic variation in the genomic region near the lead SNP of TRAV1-2 (rs2256752), but are driven by different causal variant loci (H3 = 99.3%). Similar to the colocalization results, SNP loci within the TRAV1-2 core region exhibited little association with either phenotype, with rs2229094 showing the strongest association, and the TRAV1-2 eQTL and PTB GWAS shared causal variants but with limited association (Figure 3M).

### 2.3. Downstream Function of CD4^+^ T_EM_ Cluster Core Markers in PTB

To validate the accuracy of the SNP predictions, we analyzed the traits associated with the lead SNPs. Although no association with PTB was reported, phenoscanner traits associated with some respiratory immune functions, such as asthma and chronic obstructive pulmonary disease, were observed (Table S3, *p* < 0.01). Steiger filtering was then performed to compare the *r*^2^ values between SNPs of exposure and SNPs of outcome, based on regional association analysis. Our reverse MR (R-MR) results (Table S4, *p* < 0.01) showed that SNPs of core markers on the causal chain were closer to the lead SNPs than SNPs of PTB.

**Figure 4:**
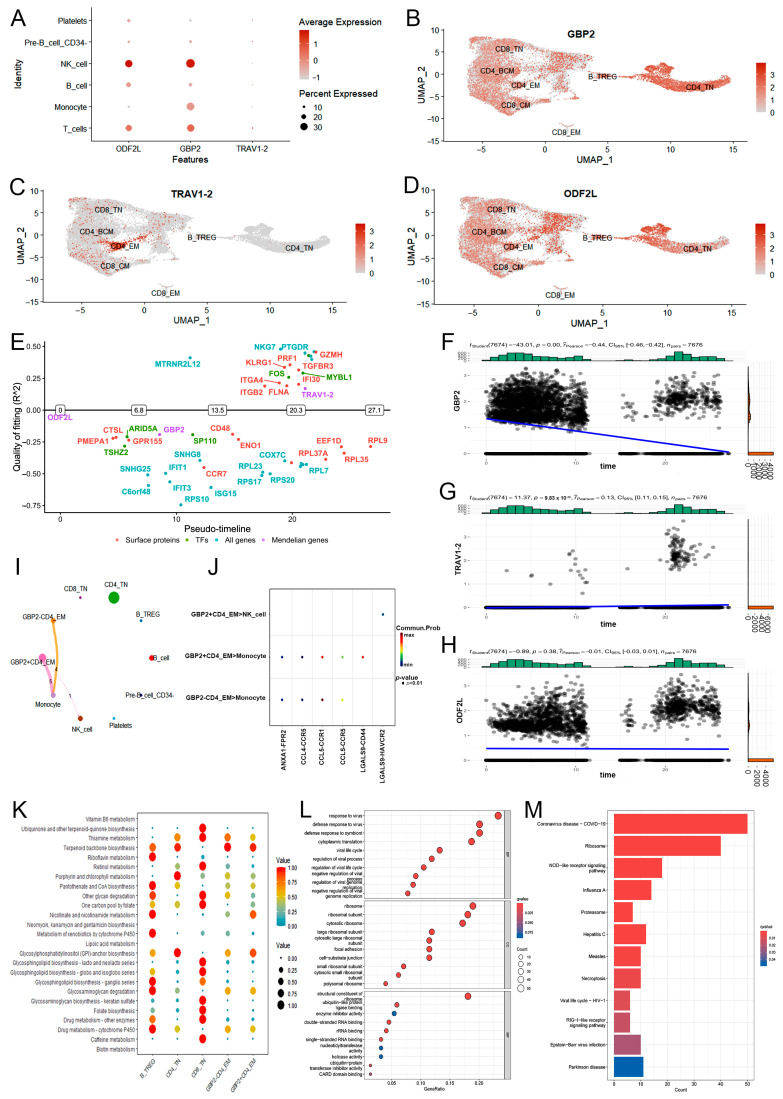
Downstream functional analysis of CD4^+^ T_EM_ cluster core markers in PTB. (**A**–**D**) The spatial distribution of marker expression in each cell (**A**) and T cell subset (**B**–**D**) is named and visualized. (**E**) Visualizing the sequential arrangement of highly dynamic genes among diverse sets of established proteins and two markers. (**F**–**H**) Expression of GBP2 (**F**), TRAV1-2 (**G**), and ODF2L (**H**) from (**E**). (**I**,**J**) The number of pairings of receptors and ligands is shown by the cell-cell communication network between GBP2-positive and -negative CD4^+^ T_EM_ cells and other T cell subsets. The thickness of each line represents the number of pairings. (**K**) Bubble diagram of enriched metabolic pathways referring to the metabolic differences between GBP2-positive and -negative CD4^+^ T_EM_ cells. Each bubble denotes a single metabolic pathway. The *p*-value and total number of metabolites engaged are listed on the right. (**L**,**M**) GO and KEGG analyses of GBP2-positive and -negative CD4^+^ T_EM_ cells.

To understand the regulatory mechanisms of core markers in CD4^+^ T_EM_ cell activation, the single-cell data were resolved. NK cells share a similar progenitor with T cells, and numerous surface molecules are shared between NK cells and T cells [18]. In this study, three core markers (GBP2 in particular) were specifically expressed by monocytes (Figure 4A). TRAV1-2 was specifically expressed by the CD4^+^ T_EM_ cell cluster of an earlier T cell stage (Figure 4B–D). Potential switch genes (surface proteins, transcription factors, and all genes) were screened for the presence of both on- and off-expression states by binarizing the genes in the differentiation trajectory. Pseudotime analysis was used to visualize the relationship between the switch time points of the top switch genes and three core markers (Figure 4E). In particular, TRAV1-2 was expressed in the late stage of CD4^+^ T_EM_ cells, whereas ODF2L and GBP2 were expressed in the early stage, corresponding to numerous switch genes (FLNA, NME2, and PLAAT4). As CD4^+^ T_EM_ cells developed, the expression of TRAV1-2 and ODF2L was upregulated, and that of GBP2 was downregulated (Figure 4F–H).

In terms of cell communication, GBP2^+^ CD4^+^ T_EM_ cells specifically interacted with NK cells via the LGALS9-HAVCR2 pathway, in contrast to GBP2^−^ CD4^+^ T_EM_ cells (Figure 4I,J). In the LGALS9-CD-45 pathway, NK cells specifically interacted with ODF2L^+^ CD4^+^ T_EM_ cells. TRAV1-2^−^ CD4^+^ T_EM_ cells communicated predominantly with monocytes via the CCL5-CCR1 pathway, whereas no TRAV1-2^+^ CD4^+^ T_EM_ cells were detected (Figure S11A–D). Immune cell activation not only causes modifications to cellular metabolism, but immune and metabolic pathways are mutually correlated [19]. GBP2^+^ CD4^+^ T_EM_ cells specifically activated glycosylphosphatidylinositol (GPI)-anchor biosynthesis in GBP2^+^ CD4^+^ T_EM_ cells but not in GBP2^−^ CD4^+^ T_EM_ cells, other T cells clusters, or ODF2L^+^ CD4^+^ T_EM_ cells (a risk factor for PTB) (Figure 4K). Results of GO and KEGG analyses showed enrichment of mononuclear cell differentiation, regulation of T cell activation, defense response to the virus, and COVID-19 in CD4^+^ T_EM_ cells with positivity for three markers (Figure 4L,M, and Figure S12).

These above results suggest that CD4^+^ T_EM_ cell clusters may act as hubs in the pathogenesis for PTB and COVID-19 infection, which was verified by MR analysis in PTB. Therefore, we examined the role of CD4^+^ T_EM_ cell clusters in COVID-19 in more detail.

### 2.4. MR Analysis of CD4^+^ T_EM_ Cluster Markers and COVID-19

**Figure 5:**
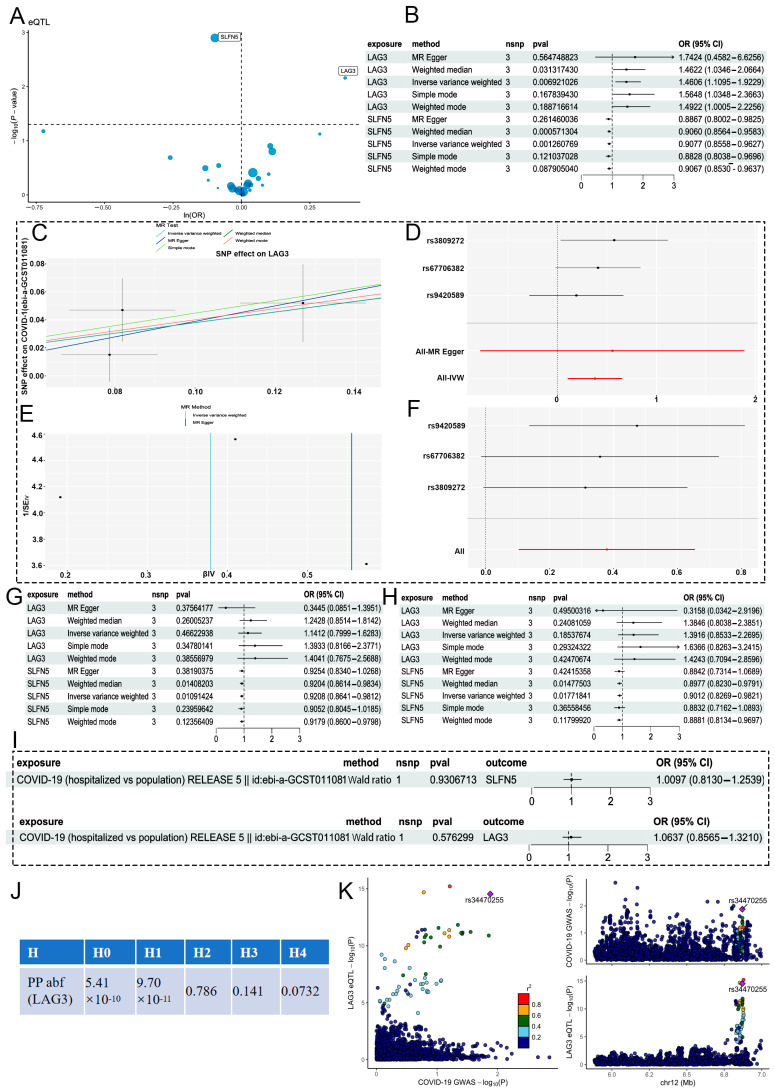
MR analysis of CD4^+^ T_EM_ cluster markers and COVID-19. (**A**) Volcano plot of the MR analysis findings for three markers on the potential risk of COVID-19 determined using the IVW method. ORs for increased risk of COVID-19 are expressed as per standard deviation increase in marker level. (**B**) Forest plot of five MR model results for the two markers. (**C**) Scatter plot of the five MR models. Each point represents an IV, the line on each point represents the 95% CI, the abscissa represents the impact of the SNP on exposure, and the ordinate represents the impact of the SNP on outcome. (**D**) Forest plot of MR analysis results for the single-SNP estimation of LAG3. The red line represents the pooled results for all SNPs. (**E**) Funnel plot of three SNPs identified by MR analysis. The red line represents the pooled results for all SNPs. (**F**) MR sensitivity results for LAG3 after removing SNPs using the leave-one-out method. (**G**,**H**) Forest plot of MR validation through ebi-a-GCST011082 (**G**) and ebi-a-GCST011075 (**H**). (**I**) Forest plot of R-MR analysis. (**J**) Results of colocalization analyses, with posterior probability. (**K**) Regional association plots of GWAS results and marker-eQTLs at markers, COVID-19, and COVID-19 locus. SNPs are colored based on LD (*r*^2^) with the lead marker-eQTL (rs67706382). Purple diamonds represent the lowest *p*-value for each locus.

MR analysis results suggested that two markers of CD4^+^ T_EM_ cells exhibit a direct causal relationship with the development of COVID-19 (Figure 5A). The exposure factors and IVs considered are listed in Table S5. Specifically, LAG3 (IVW, 3 SNPs, *p* = 0.0069) was found to be a risk factor along with the most notable SNP (rs67706382, *p* = 6.16 × 10^−16^), but SLFN5 (IVW, 3 SNPs, *p* = 0.0012) with the most notable SNP (rs67706382, *p* = 6.16 × 10^−16^) was a protective factor against COVID-19 (Figure 5B). Rs67706382 from LAG3 had the most significant *p*-value (*p* = 6.16 × 10^−16^) and maximum F statistic (*F* = 65.38), whereas rs7215469 from SLFN5 had the most significant *p*-value (*p* = 1 × 10^−200^) and maximum *F* statistic (*F* = 2796.80, Table 2), both without evidence of heterogeneity (all *p* > 0.1; Table 3), pleiotropy (all *p* < 0.01, Table 3). Scatter, forest, funnel, and leave-one-out plots for LAG3 were also generated (Figure 5C–F). Validation sets for patients of different ethnicities and varying COVID-19 severity confirmed the reliability of the two markers (Figure 5G,H). The scatter, forest, funnel, and leave-one-out plots are provided in Figure S13. Subsequently, we also examined marker commonalities between PTB and COVID-19. No reverse causality of LAG3 (*p* = 0.57, Figure 5I), or SLFN5 (*p* = 0.93, Figure 5I) were found. However, there were no significant results to indicate that GBP2, TRAV1-2, and ODF2L can be used as factors to estimate the incidence of COVID-19 (Figure S14).

Only one of these two phenotypes (LAG3 eQTL and COVID-19) was genetically linked around rs67706382 (PP abf of H2 = 73.6%, Figure 5J). SNP loci of the LAG3 core region were associated with both of these phenotypes, with the strongest association for rs34470255 (Figure 5K).

### 2.5. Downstream Function of CD4^+^ T_EM_ Cluster’s Core Markers in COVID-19

**Figure 6:**
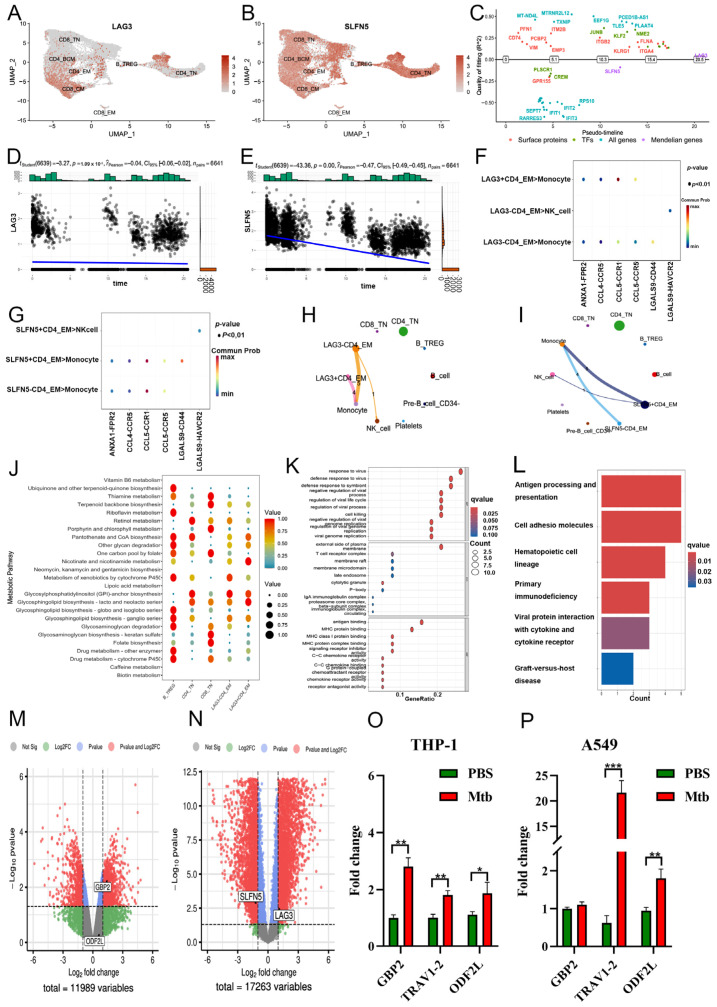
Downstream functional analysis of CD4^+^ T_EM_ cluster core markers in COVID-19. (**A**,**B**) The spatial distribution of marker expression in each cell subset is designated and visualized using UMAP. (**C**) An illustration of the most prominent switching gene order derived from two markers and different sets of known proteins. (**D**,**E**) Expression of LAG3 (**D**) and SLFN5 (**E**) from (**C**). (**F**–**I**) The number of pairings of receptors and ligands between marker-positive and -negative CD4^+^ T_EM_ cells and other T cell subsets is shown by the cell-cell communication network. LAG3 (**F**,**H**), SLFN5 (**G**,**I**). The thickness of each line represents the number of pairs. (**J**) Enrichment of metabolic pathways by a bubble diagram founded on differential metabolites between marker-positive and -negative CD4^+^ T_EM_ cells. Each bubble denotes a single metabolic pathway. The number of involved metabolites and the *p*-value are listed on the right. (**K**,**L**) GO and KEGG analysis of GBP2-positive and -negative CD4^+^ T_EM_ cells. (**M**,**N**) Volcano plots of differences in expression of PTB (**M**) and COVID-19 (**N**) CD4^+^ T_EM_ cell markers detected using bulk RNA-seq analysis. (**O**,**P**) The mRNA expression level of CD4^+^ T_EM_ cell markers (GBP2, TRAV1-2, and ODF2L) in the THP-1 macrophages (**O**) and A549 (**P**) infected with *M. tuberculosis*. Data are represented as averages ± SD. * *p* <0.05, ** *p* <0.01, *** *p* <0.001.

Some respiratory immune effects, such as asthma, were associated with these lead SNPs in phenoscanner trait analyses (Table S6, all *p* < 0.01). Compared with SNPs associated with COVID-19, these lead SNPs on the causal chain were closer to the lead SNPs, consistent with our R-MR analysis results (Table S7, all *p* < 0.01). Similar to TRAV1-2, LAG3 was specifically expressed in CD4^+^ T_EM_ and CD8^+^ T_CM_ clusters during an earlier T cell stage. However, SLFN5 did not demonstrate similar T-cell specificity (Figure 6A,B). Furthermore, pseudotime was used to visualize the relationship between the switch times of top switch genes and these two core markers. Three switch genes (GTF3A, FLNA, and PLAAT4) had a switch state similar to LAG3 (Figure 6C). As CD4^+^ T_EM_ cells developed, the expression of both LAG3 and SLFN5 in COVID-19 was upregulated (Figure 6D,E).

Regarding cell communication, LAG3^+^ CD4^+^ T_EM_ cells communicated predominantly with monocytes via the CCL5-CCR1 pathway and with NK cells via the LGALS9-HAVCR2 pathway, in contrast to CD4^+^ T_EM_ cells (Figure 6F,G). SLFN5^+^ CD4^+^ T_EM_ cells exhibited similar results to LAG3^+^ CD4^+^ T_EM_ cells (Figure 6H,I). Metabolically, both LAG3^+^ CD4^+^ T_EM_ and SLFN5^+^ CD4^+^ T_EM_ cells specifically activated nicotinate and nicotinamide metabolism, but not in LAG3^−^ CD4^+^ T_EM_ cells, SLFN5^−^ CD4^+^ T_EM_ cells, and other T cell clusters (Figure 6J and Figure S12). Results of GO and KEGG analyses showed that response to the virus, focal adhesion, cadherin binding, and COVID-19 were enriched in CD4^+^ T_EM_ cells with two positive markers (Figure 6K,L and Figure S12). The expression levels of markers were validated in the peripheral blood bulk RNAseq data of PTB (Figure 6M) and COVID-19 patients (Figure 6N). 

Finally, the mRNA expression levels of GBP2, TRAV1-2, and ODF2L were significantly elevated after *M. tuberculosis* stimulation in THP-1 macrophage (Figure 6O), with TRAV1-2 upregulated in both THP-1 and A549 cells (Figure 6P).

Our above results suggest that CD4^+^ T_EM_ cell clusters may act as hubs in the pathogenesis for PTB and COVID-19 infection, which were verified by MR analyses.

## 3. Discussion

**Figure 7:**
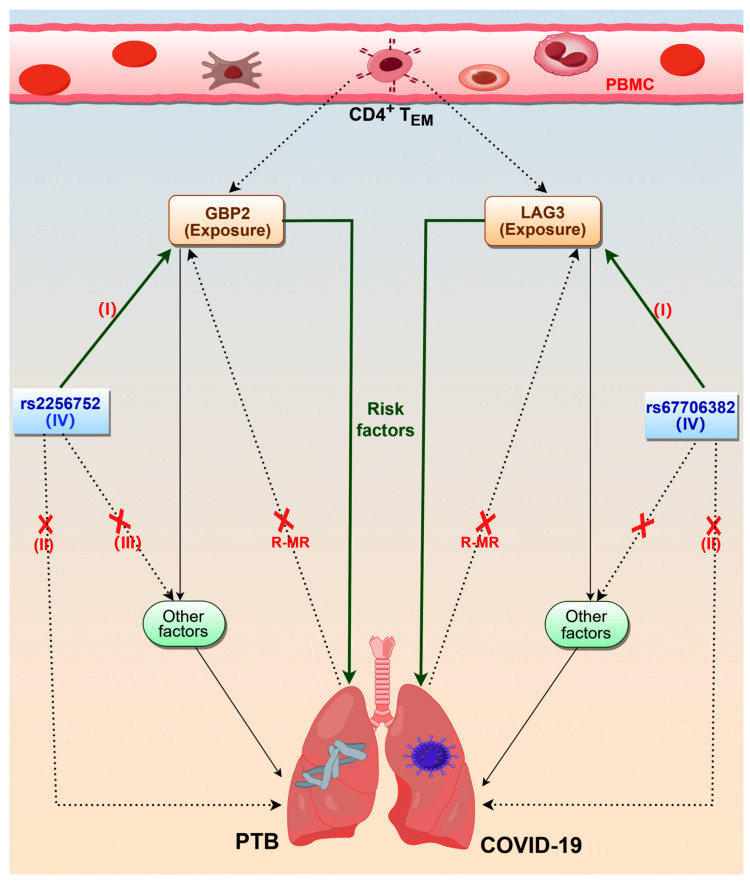
The causality of CD4^+^ T_EM_ markers and PTB and COVID-19 infection based on MR analysis (by Figdraw 2.0). I = instrumental variable relevance: IV is associated with the marker’s expressive level. II = no direct effect: IV affects the risk of PTB or COVID-19 via exposure, not through other pathways. III = instrumental variable independence: IV is not associated with either known or unknown confounders). IV = lead SNP of instrumental variables. R-MR = reverse mendelian randomization. Notes: The combination of dashed lines and cross implies no correlation. These solid green lines represent our conclusion.

This study aimed to investigate the commonality between PTB and COVID-19 based on scRNA-seq and eQTL analyses and shed new light on the molecular etiology of PTB and COVID-19 infection. We identified an important cell subset of PBMCs, the CD4^+^ T_EM_ cell cluster, that is specific and exhibits similar functions in PBMCs from both PTB and COVID-19 patients. A two-sample MR analysis was performed to evaluate the causative influence of CD4^+^ T_EM_ cell markers on PTB and COVID-19 utilizing the biggest GWAS summary-level data publicly accessible to date. Our findings revealed a causal impact of CD4^+^ T_EM_ cell markers on the risk of PTB and COVID-19, providing evidence identifying two markers (GBP2 and LAG3) as prospective targets for the prevention of PTB and COVID-19 infection (Figure 7). This is the first study examining the association of PBMC markers with PTB and COVID-19 infection using MR methods.

We identified the marker eQTLs, which explain how genetic differences maintain tissue homeostasis and target *MTB* and SARS-CoV-2 through immune variation at the cellular level. The PBMCs serve as a crucial component in the immune system, and an imbalance in PBMC homeostasis is a major cause of PTB [20] and COVID-19 [21]. The fluctuations of PBMC subsets were more dramatic in patients with COVID-19 as an acute infectious disease compared with PTB, a chronic infectious disease (Figure S3B). Rather than contrast the differences between the PTB and COVID-19 single-cell data, we focused on immune cell homogeneity by integrating the two datasets. CD4^+^ T_EM_ cells, representing effector memory CD4^+^ T cells, have received extensive attention in anti-infection research. CD4^+^ T_EM_ cell clusters represent an important component of the immune response and antigen clearance system, as they generate rapid and efficient immune responses against foreign organisms and xenoantigens [22]. Increased numbers of CD4^+^ T_EM_ cells have been reported in infectious illnesses involving PTB [23] and COVID-19 [24], and this is thought to be a novel observational indicator of clinical diagnosis and outcome. We focused on CD4^+^ T_EM_ cell clusters not only for elevated expression levels but also functionally for intercellular communication, primarily with monocytes and B cells. Researchers have reported that effector memory T (T_EM_) cells are rapidly transformed from central memory T (T_CM_) cells and generate inflammatory factors, including IFN-γ and IL-17, to exert effector functions after antigen stimulation and persist in the periphery [25,26]. CD4^+^ T_EM_ cells are more effective in enhancing B-cell function to synthesize antibodies more rapidly to protect against pathogens than naïve CD4^+^ T cells [27]. Furthermore, the formation of primary responding B cell germinal centers and secretion of high-affinity class-switched antibodies are CD4^+^ independent [28]. It is recognized that interactions between CD4^+^ T cells and monocytes promote both pro- and anti-inflammatory responses [29], which is also observed in marker-positive cellular communication analyses. Enrichment analyses also demonstrated a connection between markers of CD4^+^ T_EM_ cells, the T cell receptor, and COVID-19. Therefore, we hypothesized that core markers of CD4^+^ T_EM_ cells may be important in driving PTB and COVID-19 infection.

MR analysis confirmed the scRNA-seq inference. Due to the lack of co-morbid GWAS datasets for PTB and COVID-19, we performed MR analyses of PTB and COVID-19 separately. We focused on the significant causality of GBP2 and LAG3 for PTB and COVID-19, respectively. The odds ratio (OR) for GBP2 in PTB was 1.38 (95% confidence interval (CI): 1.05–1.81), suggesting that patients with GBP2^+^ CD4^+^ T_EM_ cells are 1.38 times more likely to develop PTB and COVID-19 infection than patients with GBP2^−^ CD4^+^ T_EM_ cells. The OR for LAG3 in COVID-19 was 1.46 (95% CI: 1.11–1.92), meaning that patients with LAG3^+^ CD4^+^ T_EM_ cells are 1.46 times more likely to develop PTB and COVID-19 infection than patients with LAG3^−^ CD4^+^ T_EM_ cells. Other studies demonstrated that GBP2 is aberrantly overexpressed in TB [30,31] and reduced in pleural tuberculosis patients after anti-TB treatment [31]. Interestingly, GBP2 is upregulated during SARS-CoV-2 infection [32] and inhibits the spike-mediated infectivity of the Wuhan-Hu-1 and Omicron variants, but not that of the Alpha and Delta variants [33]. Another study that analyzed the immunophenotype of blood found that activation of LAG3, an immunosuppressive factor, was responsible for immune dysregulation in COVID-19 patients [34]. An immune-suppressive environment characterized by an increasing number of LAG3-expressing CD4^+^ T cells in the lung increases the susceptibility of aged mice to *MTB* infection [35]. These results explain why these markers described here are risk factors for PTB and COVID-19 infection but originate from CD4^+^ T_EM_ cells that contribute to the defense against pathogen infection. Here, we demonstrated this using R-MR and Steiger analyses. These two analyses showed that the markers are neither risk nor protective factors for PTB or COVID-19. These data suggest that these three markers do not play a protective role in the immune response to pathogen invasion or in slowing down the disease process in the initial phases of PTB and COVID-19 development. For sensitivity analyses, MR-Egger intercepts and MR-PRESSO did not indicate the presence of average pleiotropic bias. Leave-one-out MR estimates did not identify any single SNPs that would drive the pooled IVW estimates. Therefore, the MR analyses in this study were not affected by bias. Furthermore, there was no causal relationship between markers of PTB and COVID-19, suggesting heterogeneity of markers of CD4^+^ T_EM_ cells across diseases.

Downstream functional results explain the mechanisms of these core marker eQLTs more specifically. We used an online search and Phenoscanner methods to explore whether these core marker eQLTs have been studied in PTB. However, these have not been found in PTB. Indeed, over 100 candidate genes associated with TB susceptibility have been identified, but few associations have proven reproducible [36], possibly due to differences in data sources. GeneSwitch identified the temporal relationship between markers and switch gene expression in the pseudotime trajectory, providing a more comprehensive understanding of the regulation of markers and the sequence of switch gene expression that occurs during the transition of CD4^+^ T_EM_ cells. As CD4^+^ T_EM_ cells develop, the alterations in marker expression are in line with the overall findings, specifically those regarding GBP2, ODF2L, LAG3, and SLFN5. An increasing number of immune metabolites, including GPI, have been identified as important regulators of immune cell function [37]. The bubble diagrams of differential metabolic pathways revealed that variations in metabolically related clusters of metabolites were driven by the expression of specific markers.

Our study has several significant advantages. In contrast to observational studies, reverse causality and confounding effects were avoided by utilizing random assignment of genetic variants as a tool in the MR analyses. In comparison with the bulk data, the utilization of scRNA-seq data allows for revealing complex and rare cell populations, gene-to-gene and cell-to-cell regulatory relationships, and tracking the trajectories of different cellular lineages during differentiation. Our results also hold practical significance. CD4^+^ T_EM_ cells and the associated markers provide clues that could help prevent and treat PTB and COVID-19 infection. Future studies for PTB and COVID-19 infection may therefore focus on CD4^+^ T_EM_ cells and their markers.

Our study also has certain limitations. Regarding data sources, the research was restricted by the small sample size for scRNA-seq analyses, especially about PTB (GSE218065). The inclusion of more subjects in future studies will be necessary to increase the results’ statistical validity. For MR analysis results, sensitivity studies such as MR-Egger and MR-PREESO were not suitable, as there were too few independently correlated SNPs found to be causally associated with PTB or COVID-19.

## 4. Materials and Methods

### 4.1. Study Overview

We used a cell cluster markers-based, two-sample MR method to evaluate the possible causation of T cell marker eQTL on PTB and COVID-19. The MR design was built on three assumptions: (1) genetic variants are strongly related to the exposure of interest (CD4^+^ T_EM_ cell markers); (2) genetic variants exhibit no correlation with potential confounding factors; (3) genetic variants demonstrably influence the outcome; (PTB and COVID-19) only through the exposure of interest. All bioinformatics analyses were carried out with the R program (version 4.1.3).

### 4.2. scRNA-Seq Data Processing

Unsupervised clustering of scRNA-seq cells was executed utilizing the “Seurat” package of R (version 4.1.1). The detection of outliers was conducted based on gene counts, unique molecular identifier counts, and percentages of mitochondrial genes. Cells with a maximum gene count of >90% or <200 were discarded. Cells with >7.5% mitochondrial genes were also assumed to be of poor quality and discarded. Genes were filtered out if they were expressed in fewer than two cells. Cells with a maximum gene count of >90%, a minor gene count of <200, or a mitochondrion proportion of >10% were discarded. Highly variable genes were calculated using the “find variable genes” method of the “Seurat” package of R, and 2000 of the calculated genes were subjected to further analyses, including PCA.

Following filtration, the batch correction was performed utilizing the “Harmony” package of R (version 1.2.0). Subsequently, the k-nearest neighbor graph was constructed based on Euclidean distances within the subspace defined by the top 10 significant principal components. The clustering of cells depicted in the image was achieved through the application of the Louvain modularity optimization algorithm. Then, the UMAP method was used for cell cluster identification. The “bimod” test, employed through the “Seurat Find Markers” method within the “Seurat” package of R, was utilized to compute the variance in expression across each cluster. Markers exhibiting a log2 average differential expression of 0.585 and statistical significance with *p* < 0.05 were discerned. Subsequently, cell clusters were annotated by leveraging canonical markers associated with well-defined cell types. The batch was then corrected using the “Harmony” package of R. The “Seurat” package of R was utilized to extract T cell subsets. After dimensionality reduction and clustering, the “reshape2” (version 1.4.4) and “ggplot2” (version 3.4.4) packages of R were utilized to calculate the proportion for each T cell subset. In combination with the researched literature [38] and the Cell Marker database [39], the cell types in the T cell clusters were annotated.

### 4.3. scRNA-Seq Data-Based Functional Analyses

To determine CD4^+^ T_EM_ cell lineages and developmental distances, the “Slingshot” package of R (version 2.10.0) [40] was used to execute the cell lineage inference algorithm and identify the lineage trajectories and bifurcations to organize CD4^+^ T_EM_ cells through them. Briefly, slingshot calculates lineage trajectories and branch points by linking cluster medoids with a minimal spanning tree and determining the initial cluster or root node.

The communication network between CD4^+^ T_EM_ cells and other PBMCs in our dataset was inferred, analyzed, and visualized with the “CellChat” package of R (version 1.6.1). In principle, CellChat takes gene expression data from cells as an input file and groups cells by constructing shared neighborhood maps based on cell distances in a pseudotemporal trajectory space. CellChat then simulates the probability of cell-cell communication based on the interactions between gene expression and signaling ligands, inter-receptors, and other cofactors. The expression distribution of markers in cell clusters was annotated using the “Viridis” package of R (version 0.6.4).

Ordering gene expression patterns and functional occurrences in single-cell experiments were carried out using the “GeneSwitches” package of R (version 0.1.0). First, binary analysis of genes in differentiation trajectories was used to search for putative switch genes having both on and off states in expression properties using GeneSwitches. Subsequently, logistic regression analyses were performed, and McFadden’s Pseudo *r*^2^ fitted temporal correlation analysis was conducted on the potential switch genes. Specifically, the switch time point for each switch gene was deduced via logistic regression analysis. Each correlation *r*^2^ value was obtained from the proposed temporal correlation analysis, in which positive correlation of the expression of activated switch genes with the proposed temporal sequence (*r*^2^ > 0) was defined as an upregulated switch gene, whereas negative correlation of the expression of silenced switch genes with the proposed temporal sequence (*r*^2^ < 0) was defined as a downregulated switch gene. Finally, the top switch genes were visualized based on their switching times and sorted according to the proposed chronology.

Metabolite set enrichment analysis (MSEA) for differential metabolic features was carried out with the “scMetabolism” package of R (version 0.2.1). To predict gene functions, we first identified markers for marker-positive and marker-negative cell subsets using the “FindAllMarkers” R package, and then GO and KEGG analyses were performed using the “clusterProfiler” (version 4.10.0) and “ggplot2” packages of R. Significance in the correlation of gene function, as determined based on the GO database and KEGG pathway, was recognized at *p* < 0.05. Volcano plots were created using the “ggrepel” package of R (version 0.9.4).

### 4.4. Instrumental Variable (IV) Screen

To provide valid IVs in the MR analysis, the IVs selected for exposure (CD4^+^ T_EM_ cell markers) should fulfill the following conditions: (1) All SNPs must demonstrate linkage equilibrium, characterized by pairwise r^2^ values of ≤0.001; (2) F statistic > 10 is required for sufficient strength to limit the bias from weak IVs; and (3) SNPs exhibit robust associations with exposure, reaching the threshold of genome-wide significance (*p* < 1 × 10⁻^5^). The formula *F* = *r*^2^ × (*N* − *k* − 1)/[(1 − *r*^2^) × *k*] is utilized, where N represents the sample size of CD4^+^ T_EM_ cell markers GWAS, k signifies the number of SNPs, and R^2^ denotes the proportion of variability in the expression of marker status explained by each SNP. R^2^ is further defined as 2 × *beta*^2^ × (1 *− EAF*) × *EAF*, where EAF represents the effect of allele frequency and beta represents the estimate of the genetic effect of each SNP on CD4^+^ T_EM_ cell markers).

### 4.5. MR Analyses

The MR design was according to the STROBE-MR Statement [41], and the MR STROBE Checklist was listed in the supplementary file. MR analyses rely on three key assumptions: (1) IV relevance (IVR): the genetic variants utilized as instruments are related to the marker eQTLs and influence the PTB; (2) IV independence (IVI): the genetic variant is unaffected by any confounding factors that might influence the relationship between the exposure and the outcome; and (3) no direct effect: there is no direct effect of the genetic variant on the outcome, other than its influence on exposure.

We used the “TwoSampleMR” package of R (version 0.5.8) to carry out several MR approaches, namely IVW, WM, weighted model, sample mode, and MR-Egger, to determine MR estimates of markers eQTL for PTB and COVID-19 after harmonizing the EAs across the GWASs of PTB or COVID-19 and markers eQTL. To analyze both conservative and liberal, the IVW approach was utilized for the primary MR estimations to assess the influence of each eQTL marker measure on the potential risk of PTB and COVID-19. Four distinct types of plots were crafted for visual examination: (1) a leave-one-out plot designed to test SNP outliers; (2) a funnel plot illustrating horizontal pleiotropy; (3) a forest plot exhibiting individual SNP effects in the MR analysis; and (4) a scatter plot facilitating a comprehensive evaluation of effect sizes in the GWAS for both the cause and outcome. In addition, the MR-PRESSO method was used to detect and correct for potential outliers.

### 4.6. R-MR Analyses

R-MR analyses were performed for all associations that survived multiple tests to investigate reverse causation (the likelihood that genetic predisposition to PTB or COVID-19 influences the marker eQTL). To further prove this, the MR Steiger directionality test was used to assess the directionality of associations between markers eQTL and PTB or COVID-19 [42].

### 4.7. Colocalization Analysis

Colocalization analysis of the GWAS and eQTL signals was performed using the “Coloc” package of R (version 5.2.3). Under Coloc, eQTL and GWAS in a Bayesian framework recognized PTB or COVID-19 GWAS signals that colocalized with markers eQTL using default parameter values and a colocalization before *P*_12_ = 10^−6^. Coloc calculates the posterior probability of association with either trait (H0), association with gene expression (H1), association with the trait (H2), association with both phenotypes but separate causal variants (H3), and association with both phenotypes sharing the same causative variant (H4). Regions with evidence for colocalization between gene expression and trait were defined as PH3 + PH4 ≥ 0.90 and PH4/PH3 ≥ 3 [43]. Colocalization of markers eQTL, markers, PTB, and COVID-19 GWAS associations was visualized by the “Locuscompare” package of R (version 1.0.0). Diseases associated with core SNP loci of markers were screened using the Phenoscanner method.

### 4.8. Cell Culture and PCR Analysis

The THP-1 cell line was obtained from Haixing Biosciences (Suzhou, China) and cultured in a complete medium containing RPMI 1640 medium (Gibico, Billings, MT, USA), with 10% fetal bovine serum (Gibico, Billings, MT, USA) and 1% penicillin-streptomycin (Manassas, VA, USA). The PMA (200 ng/mL) was used to induce the differentiation of THP-1 cells into macrophage-like cells, thereby augmenting their phagocytic capabilities. After 48 h, noticeable changes in cell morphology were observed, including adherence to the culture plates and the adoption of shapes categorized as oval, round, or irregular, indicative of macrophage characteristics.

A549 cell line was purchased from ATCC (Manassas, VA, USA) and cultured in a complete medium containing DMEM medium (Gibico, Billings, MT, USA), with 10% fetal bovine serum (Gibico, Billings, MT, USA) and 1% penicillin-streptomycin (Manassas, VA, USA).

All cells were infected with *MTB* at a MOI of 10 (*MTB*: cells) for 6 h. After infection, the total RNA was collected by a Trizol kit (Invitrogen Inc., Carlsbad, CA, USA), whose RNA was reverse transcribed into cDNA (TaKaRa, Kusatsu-shi, Japan). QPCR was then conducted with TB Green Premix Ex Taq II (TaKaRa, RR820, Kusatsu-shi, Japan) using the Bio-Rad CFX96 real-time PCR system, with β-actin serving as an internal control. The PCR primers are shown in Table 4.

## 5. Conclusions

This study based on sc-eQTL and MR analyses provides evidence for a causal effect of CD4^+^ T_EM_ markers on PTB and COVID-19, separately. Notably, GBP2 is causally linked to PTB, serving as a risk factor. LAG3 functions as a risk factor for COVID-19. Our result sheds light on the changes in peripheral blood T cell subsets between PTB and COVID-19 infection. CD4^+^ T_EM_ markers will be invaluable in ultimately understanding the common pathogenesis of PTB and COVID-19 and in developing more precise diagnostic and therapeutic molecular targets for clinical practice.

## Figures and Tables

**Table 1:** Details of the datasets included in this study.

Dataset	Source	Characteristic
PTB ScRNA-seq	GSE218065 https://www.ncbi.nlm.nih.gov/geo/query/acc.cgi?acc=GSE218065 (accessed on 13 December 2023)	Male, PBMC
PTB ScRNA-seq	GSE192483 https://www.ncbi.nlm.nih.gov/geo/query/acc.cgi?acc=GSE192483 (accessed on 13 December 2023)	Lung tissue
COVID-19 scRNA-seq	GSE171555 https://www.ncbi.nlm.nih.gov/geo/query/acc.cgi?acc=GSE171555 (accessed on 13 December 2023)	Male, PBMC
COVID-19 scRNA-seq	GSE192391 https://www.ncbi.nlm.nih.gov/geo/query/acc.cgi?acc=GSE192391 (accessed on 13 December 2023)	PBMC
PTB bulk	GSE65517 https://www.ncbi.nlm.nih.gov/geo/query/acc.cgi?acc=GSE65517 (accessed on 5 February 2024)	Male, PBMC
COVID-19 bulk	GSE215262 https://www.ncbi.nlm.nih.gov/geo/query/acc.cgi?acc=GSE215262 (accessed on 5 February 2024)	PBMC
PTB GWAS	ebi-a-GCST90018892 https://gwas.mrcieu.ac.uk/datasets/ebi-a-GCST90018892/ (accessed on 2 January 2024)	477,386 sample, European
PTB validation-1 GWAS	ebi-a-GCST90018672 https://gwas.mrcieu.ac.uk/datasets/ebi-a-GCST90018672/ (accessed on 2 January 2024)	178,671 sample, East Asian
PTB validation-2 GWAS	bbj-a-149 https://gwas.mrcieu.ac.uk/datasets/bbj-a-149/ (accessed on 2 January 2024)	212,453 sample, East Asian, Males, and Females
PTB validation-3 GWAS	finn-b-TBC_RESP https://gwas.mrcieu.ac.uk/datasets/finn-b-TBC_RESP/ (accessed on 2 January 2024)	European, Males, and Females
COVID-19 GWAS	ebi-a-GCST011081 https://gwas.mrcieu.ac.uk/datasets/ebi-a-GCST011081/ (accessed on 10 January 2024)	1,887,658 sample, European
COVID-19 validation-1 GWAS	ebi-a-GCST011082 https://gwas.mrcieu.ac.uk/datasets/ebi-a-GCST011082/ (accessed on 10 January 2024)	1,557,411 sample, European
COVID-19 validation-2 GWAS	ebi-a-GCST011075 https://gwas.mrcieu.ac.uk/datasets/ebi-a-GCST011075/ (accessed on 10 January 2024)	1,388,342 sample, European, severe COVID-19

Notes: scRNA-seq (single-cell RNA sequencing); GWAS (genome-wide association study).

**Table 2:** MR estimates of markers.

Marker	Ensemble ID	Outcome	SNP	EA	OR (95% CI)	*p* Value	PVE	*F* Statistic
ODF2L	ENSG00000122417	PTB	rs7523135	G	0.94 (0.89–1.00)	0.041	10.36%	2597.99
ODF2L	ENSG00000122417	PTB	rs6576834	C	0.94 (0.89–1.00)	0.041	10.36%	670.87
ODF2L	ENSG00000122417	PTB	rs5744305	G	0.94 (0.89–1.00)	0.041	10.36%	118.33
ODF2L	ENSG00000122417	PTB	rs61161313	T	0.94 (0.89–1.00)	0.041	10.36%	40.94
ODF2L	ENSG00000122417	PTB	rs4512701	A	0.94 (0.89–1.00)	0.041	10.36%	40.92
GBP2	ENSG00000162645	PTB	rs2182322	G	1.38 (1.05–1.81)	0.0208	1.60%	47.99
GBP2	ENSG00000162645	PTB	rs76830965	A	1.38 (1.05–1.81)	0.0208	1.60%	40.69
GBP2	ENSG00000162645	PTB	rs9264638	A	1.38 (1.05–1.81)	0.0208	1.60%	52.07
GBP2	ENSG00000162645	PTB	rs149007767	T	1.38 (1.05–1.81)	0.0208	1.60%	57.79
GBP2	ENSG00000162645	PTB	rs653178	T	1.38 (1.05–1.81)	0.0208	1.60%	223.35
GBP2	ENSG00000162645	PTB	rs4788084	T	1.38 (1.05–1.81)	0.0208	1.60%	51.35
TRAV1-2	ENSG00000256553	PTB	rs13325613	T	1.37 (1.09–1.72)	0.00684	0.75%	60.48
TRAV1-2	ENSG00000256553	PTB	rs3130559	T	1.37 (1.09–1.72)	0.00684	0.75%	40.1
TRAV1-2	ENSG00000256553	PTB	rs2256752	C	1.37 (1.09–1.72)	0.00684	0.75%	61.35
LAG3	ENSG00000089692	COVID-19	rs9420589	T	1.46 (1.11–1.92)	0.00692	0.48%	42.93
LAG3	ENSG00000089692	COVID-19	rs3809272	T	1.46 (1.11–1.92)	0.00692	0.48%	38.49
LAG3	ENSG00000089692	COVID-19	rs67706382	A	1.46 (1.11–1.92)	0.00692	0.48%	65.38
SLFN5	ENSG00000166750	COVID-19	rs7215469	A	0.91 (0.86–0.96)	0.00126	9.10%	2796.81
SLFN5	ENSG00000166750	COVID-19	rs76240782	T	0.91 (0.86–0.96)	0.00126	9.10%	256.05
SLFN5	ENSG00000166750	COVID-19	rs8076768	T	0.91 (0.86–0.96)	0.00126	9.10%	48.33

Notes: SNP (single nucleotide polymorphism); EA (effect allele); OR (odds ratio); CI (confidence interval); PVE (phenotypic variance explained).

**Table 3:** Marker MR results and heterogeneity and horizontal pleiotropy tests.

Exposure	Outcome	Method	Q	*P* (Heterogeneity)	*P* (Pleiotropy)
GBP2 (eqtl-a-ENSG00000162645)	PTB (ebi-a-GCST90018892)	MR Egger	7.45	0.11	0.69
GBP2 (eqtl-a-ENSG00000162645)	PTB (ebi-a-GCST90018892)	IVW	7.80	0.17	-
TRAV1-2 (eqtl-a-ENSG00000256553)	PTB (ebi-a-GCST90018892)	MR Egger	0.24	0.63	0.45
TRAV1-2 (eqtl-a-ENSG00000256553)	PTB (ebi-a-GCST90018892)	IVW	1.58	0.45	-
ODF2L (eqtl-a-ENSG00000122417)	PTB (ebi-a-GCST90018892)	MR Egger	2.06	0.56	0.57
ODF2L (eqtl-a-ENSG00000122417)	PTB (ebi-a-GCST90018892)	IVW	2.46	0.65	-
LAG3 (eqtl-a-ENSG00000089692)	COVID-19 (ebi-a-GCST011071)	MR Egger	1.96	0.16	0.76
LAG3 (eqtl-a-ENSG00000089692)	COVID-19 (ebi-a-GCST011071)	IVW	2.28	0.32	-
SLFN5 (eqtl-a-ENSG00000166750)	COVID-19 (ebi-a-GCST011071)	MR Egger	0.52	0.47	0.97
SLFN5 (eqtl-a-ENSG00000166750)	COVID-19 (ebi-a-GCST011071)	IVW	0.52	0.77	-

**Table 4:** PCR primer sequences.

Gene	Forward (5′-3′)	Reverse (5′-3′)
GBP2	AATTAGGGGCCCAGTTGGAAG	AAGAGACGGTAACCTCCTGGT
TRAV1-2	GCTACGGAAGGTGCCATTGT	AATGTAGGTGCTTCGCCAGC
ODF2L	AAAGCAAACCGTTTTTCCCAATC	CGTTCTCGGCTTCCCTTTTATG
β-actin	CACTCTTCCAGCCTTCCTTC	GTACAGGTCTTTGCGGATGT

## Data Availability

Data can be requested from the corresponding author.

## Supplementary Materials

The following supporting information can be downloaded at: https://www.mdpi.com/article/10.3390/ijms25189971/s1.

## Abbreviations

CD4^+^ T_EM_: CD4^+^ effective memory T cell; sc-eQTL: single-cell expression quantitative trait locus; PTB: pulmonary tuberculosis; COVID-19: coronavirus disease 2019; MR: mendelian randomization; GWAS: genome-wide association study; GO: gene ontology; KEGG: kyoto encyclopedia of genes and genomes; GPI: glycosylphosphatidylinositol; PBMC: peripheral blood mononuclear cell; eQTL: expression quantitative trait locus; *MTB*: *Mycobacterium tuberculosis*; TB: tuberculosis; PCA: principal components analysis; UMAP: uniform manifold approximation and projection; IVR: IV relevance; IVI: IV independence; OR: odds ratio; CI: confidence interval; MSEA: metabolite set enrichment analysis; scRNA-seq: single-cell RNA sequencing; EA: effect allele; PVE: phenotypic variance explained; IVW: inverse variance weighted; WM: weighted median; R-MR: reverse mendelian randomization.

## References

[1] Chai Q., Lu Z., Liu C.H. (2020). Host defense mechanisms against *Mycobacterium tuberculosis*. Cell Mol. Life Sci..

[2] Bagcchi S. (2023). WHO’s Global Tuberculosis Report 2022. Lancet Microbe.

[3] Parker A., Boloko L., Moolla M.S., Ebrahim N., Ayele B.T., Broadhurst A.G.B., Mashigo B., Titus G., de Wet T., Boliter N. (2022). Clinical features and outcomes of COVID-19 admissions in a population with a high prevalence of HIV and tuberculosis: A multicentre cohort study. BMC Infect. Dis..

[4] Crisan-Dabija R., Grigorescu C., Pavel C.A., Artene B., Popa I.V., Cernomaz A., Burlacu A. (2020). Tuberculosis and COVID-19: Lessons from the Past Viral Outbreaks and Possible Future Outcomes. Can. Respir..

[5] Freund O., Azolai L., Sror N., Zeeman I., Kozlovsky T., Greenberg S.A., Epstein Weiss T., Bornstein G., Tchebiner J.Z., Frydman S. (2023). Diagnostic delays among COVID-19 patients with a second concurrent diagnosis. J. Hosp. Med..

[6] Toor S.M., Saleh R., Sasidharan Nair V., Taha R.Z., Elkord E. (2021). T-cell responses and therapies against SARS-CoV-2 infection. Immunology.

[7] Liu W.D., Wang J.T., Hung C.C., Chang S.C. (2022). Accelerated progression of pulmonary tuberculosis in a COVID-19 patient after corticosteroid treatment. J. Microbiol. Immunol. Infect..

[8] Motta I., Centis R., D’Ambrosio L., García-García J.M., Goletti D., Gualano G., Lipani F., Palmieri F., Sánchez-Montalvá A., Pontali E. (2020). Tuberculosis, COVID-19 and migrants, Preliminary analysis of deaths occurring in 69 patients from two cohorts. Pulmonology.

[9] Mousquer G.T., Peres A., Fiegenbaum M. (2021). Pathology of TB/COVID-19 Co-Infection, The phantom menace. Tuberculosis.

[10] Divangahi M., Desjardins D., Nunes-Alves C., Remold H.G., Behar S.M. (2010). Eicosanoid pathways regulate adaptive immunity to *Mycobacterium tuberculosis*. Nat. Immunol..

[11] Shafiani S., Tucker-Heard G., Kariyone A., Takatsu K., Urdahl K.B. (2010). Pathogen-specific regulatory T cells delay the arrival of effector T cells in the lung during early tuberculosis. J. Exp. Med..

[12] van de Vegte Y.J., Said M.A., Rienstra M., van der Harst P., Verweij N. (2020). Genome-wide association studies and Mendelian randomization analyses for leisure sedentary behaviors. Nat. Commun..

[13] McHenry M.L., Simmons J., Hong H., Malone L.L., Mayanja-Kizza H., Bush W.S., Boom W.H., Hawn T.R., Williams S.M., Stein C.M. (2023). Tuberculosis severity associates with variants and eQTLs related to vascular biology and infection-induced inflammation. PLoS Genet..

[14] Hong H., Dill-McFarland K.A., Simmons J.D., Peterson G.J., Benchek P., Mayanja-Kizza H., Boom W.H., Stein C.M., Hawn T.R. (2024). *Mycobacterium tuberculosis*-dependent monocyte expression quantitative trait loci, cytokine production, and TB pathogenesis. Front. Immunol..

[15] Wu Y., Zhang C.Y., Wang L., Li Y., Xiao X. (2023). Genetic Insights of Schizophrenia via Single Cell RNA-Sequencing Analyses. Schizophr. Bull..

[16] Sekine T., Perez-Potti A., Rivera-Ballesteros O., Stralin K., Gorin J.B., Olsson A., Llewellyn-Lacey S., Kamal H., Bogdanovic G., Muschiol S. (2020). Robust T Cell Immunity in Convalescent Individuals with Asymptomatic or Mild COVID-19. Cell.

[17] Nica A.C., Dermitzakis E.T. (2013). Expression quantitative trait loci: Present and future. Philos. Trans. R. Soc. Lond. B Biol. Sci..

[18] Zhu Y., Yao S., Chen L. (2011). Cell surface signaling molecules in the control of immune responses: A tide model. Immunity.

[19] Lötscher J., Balmer M.L. (2019). Sensing between reactions-how the metabolic microenvironment shapes immunity. Clin. Exp. Immunol..

[20] Habtamu M., Abrahamsen G., Aseffa A., Andargie E., Ayalew S., Abebe M., Spurkland A. (2020). High-throughput analysis of T cell-monocyte interaction in human tuberculosis. Clin. Exp. Immunol..

[21] Schulte-Schrepping J., Reusch N., Paclik D., Baßler K., Schlickeiser S., Zhang B., Krämer B., Krammer T., Brumhard S., Bonaguro L. (2020). Severe COVID-19 Is Marked by a Dysregulated Myeloid Cell Compartment. Cell.

[22] McKinstry K.K., Strutt T.M., Swain S.L. (2010). The potential of CD4 T-cell memory. Immunology.

[23] Li J., Jing Q., Hu Z., Wang X., Hu Y., Zhang J., Li L. (2023). *Mycobacterium tuberculosis*-specific memory T cells in bronchoalveolar lavage of patients with pulmonary tuberculosis. Cytokine.

[24] Al Saihati H.A., Hussein H.A.M., Thabet A.A., Wardany A.A., Mahmoud S.Y., Farrag E.S., Mohamed T.I.A., Fathy S.M., Elnosary M.E., Sobhy A. (2023). Memory T Cells Discrepancies in COVID-19 Patients. Microorganisms.

[25] Raphael I., Joern R.R., Forsthuber T.G. (2020). Memory CD4(+) T Cells in Immunity and Autoimmune Diseases. Cells.

[26] Jain A., Song R., Wakeland E.K., Pasare C. (2018). T cell-intrinsic IL-1R signaling licenses effector cytokine production by memory CD4 T cells. Nat. Commun..

[27] MacLeod M.K., David A., McKee A.S., Crawford F., Kappler J.W., Marrack P. (2011). Memory CD4 T cells that express CXCR5 provide accelerated help to B cells. J. Immunol..

[28] MacLennan I.C., Gulbranson-Judge A., Toellner K.M., Casamayor-Palleja M., Chan E., Sze D.M., Luther S.A., Orbea H.A. (1997). The changing preference of T and B cells for partners as T-dependent antibody responses develop. Immunol. Rev..

[29] Westhorpe C.L.V., Norman M.U., Hall P., Snelgrove S.L., Finsterbusch M., Li A., Lo C., Tan Z.H., Li S., Nilsson S.K. (2018). Effector CD4(+) T cells recognize intravascular antigen presented by patrolling monocytes. Nat. Commun..

[30] Corrêa R.D.S., Leal-Calvo T., Mafort T.T., Santos A.P., Leung J., Pinheiro R.O., Rufino R., Moraes M.O., Rodrigues L.S. (2023). Reanalysis and validation of the transcriptional pleural fluid signature in pleural tuberculosis. Front. Immunol..

[31] Long N.P., Phat N.K., Yen N.T.H., Park S., Park Y., Cho Y.S., Shin J.G. (2021). A 10-gene biosignature of tuberculosis treatment monitoring and treatment outcome prediction. Tuberculosis.

[32] Martin-Sancho L., Lewinski M.K., Pache L., Stoneham C.A., Yin X., Becker M.E., Pratt D., Churas C., Rosenthal S.B., Liu S. (2021). Functional landscape of SARS-CoV-2 cellular restriction. Mol. Cell.

[33] Mesner D., Reuschl A.K., Whelan M.V.X., Bronzovich T., Haider T., Thorne L.G., Ragazzini R., Bonfanti P., Towers G.J., Jolly C. (2023). SARS-CoV-2 evolution influences GBP and IFITM sensitivity. Proc. Natl. Acad. Sci. USA.

[34] Paine S.K., Choudhury P., Alam M., Bhattacharyya C., Pramanik S., Tripathi D., Das C., Patel V., Ghosh S., Chatterjee S. (2024). Multi-faceted dysregulated immune response for COVID-19 infection explaining clinical heterogeneity. Cytokine.

[35] Lafuse W.P., Wu Q., Kumar N., Saljoughian N., Sunkum S., Ahumada O.S., Turner J., Rajaram M.V.S. (2022). Psychological stress creates an immune suppressive environment in the lung that increases susceptibility of aged mice to *Mycobacterium tuberculosis* infection. Front. Cell. Infect. Microbiol..

[36] Naranbhai V. (2016). The Role of Host Genetics (and Genomics) in Tuberculosis. Microbiol. Spectr..

[37] Deng W., Zeng J., Xiang X., Xie J. (2012). Insights into the distribution and functions of the eukaryotic GPI-like anchored genes among Mycobacterium from a comparative genomic perspective. Crit. Rev. Eukaryot. Gene Expr..

[38] Zhang L., Yu X., Zheng L., Zhang Y., Li Y., Fang Q., Gao R., Kang B., Zhang Q., Huang J.Y. (2018). Lineage tracking reveals dynamic relationships of T cells in colorectal cancer. Nature.

[39] Zhang X., Lan Y., Xu J., Quan F., Zhao E., Deng C., Luo T., Xu L., Liao G., Yan M. (2019). CellMarker: A manually curated resource of cell markers in human and mouse. Nucleic Acids Res..

[40] Street K., Risso D., Fletcher R.B., Das D., Ngai J., Yosef N., Purdom E., Dudoit S. (2018). Slingshot: Cell lineage and pseudotime inference for single-cell transcriptomics. BMC Genom..

[41] Skrivankova V.W., Richmond R.C., Woolf B.A.R., Yarmolinsky J., Davies N.M., Swanson S.A., VanderWeele T.J., Higgins J.P.T., Timpson N.J., Dimou N. (2021). Strengthening the Reporting of Observational Studies in Epidemiology Using Mendelian Randomization, The STROBE-MR Statement. JAMA.

[42] Hemani G., Tilling K., Davey Smith G. (2017). Orienting the causal relationship between imprecisely measured traits using GWAS summary data. PLoS Genet..

[43] Guo H., Fortune M.D., Burren O.S., Schofield E., Todd J.A., Wallace C. (2015). Integration of disease association and eQTL data using a Bayesian colocalisation approach highlights six candidate causal genes in immune-mediated diseases. Hum. Mol. Genet..

