# Supplementary material for: CD4+ Effective Memory T Cell Markers GBP2 and LAG3 Are Risk Factors for PTB and COVID-19 Infection: A Study Integrating Single-Cell Expression Quantitative Trait Locus and Mendelian Randomization Analyses
Source: Int J Mol Sci. 2024 Sep 16;25(18):9971. doi: 10.3390/ijms25189971 (PMC11432203; doi:10.3390/ijms25189971)
Supplement: Supplementary file 1 [file ijms-25-09971-s001.zip › Supplementary material/MR Strobe Checklist.pdf]

STROBE-MR checklist of recommended items to address in reports of Mendelian randomization studies[1 2]

Item No.	Section	Checklist item	Relevant text from manuscript
1	**TITLE and ABSTRACT**	Indicate Mendelian randomization (MR) as the study's design in the title and/or the abstract if that is a main purpose of the study	Title and Abstract sections.
	**INTRODUCTION**		
2	**Background**	Explain the scientific background and rationale for the reported study. What is the exposure? Is a potential causal relationship between exposure and outcome plausible? Justify why MR is a helpful method to address the study question	Scientific background and rationale: paragraphs 1-2 in Introduction section;<br>The exposure is the marker of $CD4^+$ T_{EM} cell;<br>Potential causal relationship: paragraphs 2 in Introduction section;<br>MR is a helpful method: paragraphs 3 in Introduction section.
3	**Objectives**	State specific objectives clearly, including pre-specified causal hypotheses (if any). State that MR is a method that, under specific assumptions, intends to estimate causal effects	Paragraph 3 in Introduction section
	**METHODS**		
4	**Study design and data sources**	Present key elements of the study design early in the article. Consider including a table listing sources of data for all phases of the study. For each data source contributing to the analysis, describe the following:	
	a)	Setting: Describe the study design and the underlying population, if possible. Describe the setting, locations, and relevant dates, including periods of recruitment, exposure, follow-up, and data collection, when available.	Study design in Methods section and was visualized in the Figure 1. (Flow chart of this MR);<br>The underlying population, exposure and relevant dates were listed in Table 1. (Details of the datasets included in this study).
	b)	Participants: Give the eligibility criteria, and the sources and methods of selection of participants. Report the sample size, and whether any power or sample size calculations were carried out prior to the main analysis	Data source in Methods section and Table 1 (Details of the datasets included in this study).
	c)	Describe measurement, quality control and selection of genetic variants	Genetic instrumental variable selection: Paragraphs 2.4 in Methods section.<br>measurement, quality control of genetic variants: Paragraphs 2.5-2.7 in Methods section.

		d)	For each exposure, outcome, and other relevant variables, describe methods of assessment and diagnostic criteria for diseases	Data source in Methods section and Table 1 (Details of the datasets included in this study).
		e)	Provide details of ethics committee approval and participant informed consent, if relevant	Not relevant.
5	**Assumptions**		Explicitly state the three core IV assumptions for the main analysis (relevance, independence and exclusion restriction) as well assumptions for any additional or sensitivity analysis	The first paragraphs of the Materials and Methods section.
6	**Statistical methods: main analysis**		Describe statistical methods and statistics used	
		a)	Describe how quantitative variables were handled in the analyses (i.e., scale, units, model)	Paragraphs 2.4 selection in Methods section.
		b)	Describe how genetic variants were handled in the analyses and, if applicable, how their weights were selected	Paragraphs 2.4 selection in Methods section.
		c)	Describe the MR estimator (e.g. two-stage least squares, Wald ratio) and related statistics. Detail the included covariates and, in case of two-sample MR, whether the same covariate set was used for adjustment in the two samples	Paragraphs 2.5 selection in Methods section.
		d)	Explain how missing data were addressed	NA
		e)	If applicable, indicate how multiple testing was addressed	The second paragraphs of 2.5 selection in Methods section (a funnel plot illustrating horizontal pleiotropy).
7	**Assessment of assumptions**		Describe any methods or prior knowledge used to assess the assumptions or justify their validity	The second paragraphs of 2.5 selection in Methods section (MR-Egger intercepts, MR-PRESSO and Leave-one-out MR).
8	**Sensitivity analyses and additional analyses**		Describe any sensitivity analyses or additional analyses performed (e.g. comparison of effect estimates from different approaches, independent replication, bias analytic techniques, validation of instruments, simulations)	The second paragraphs of 2.5 selection in Methods section (forest plot and scatter plot).
9	**Software and pre-registration**			
		a)	Name statistical software and package(s), including version and settings used	Paragraphs 2.4–2.7 selection in Methods section
		b)	State whether the study protocol and details were pre-registered (as well as when and where)	NA
	**RESULTS**			

10	**Descriptive data**			
		a)	Report the numbers of individuals at each stage of included studies and reasons for exclusion. Consider use of a flow diagram	Figure 1. Flow chart of this MR
		b)	Report summary statistics for phenotypic exposure(s), outcome(s), and other relevant variables (e.g. means, SDs, proportions)	Table 2. MR estimates of markers
		c)	If the data sources include meta-analyses of previous studies, provide the assessments of heterogeneity across these studies	NA
		d)	For two-sample MR:<br>i. Provide justification of the similarity of the genetic variant-exposure associations between the exposure and outcome samples<br>ii. Provide information on the number of individuals who overlap between the exposure and outcome studies	NA (This study aims to explore the genetic connection between single-cell subsets and two diseases (PTB and COVID-19) using MR methods, which is not a traditional two-sample MR study).
11	**Main results**			
		a)	Report the associations between genetic variant and exposure, and between genetic variant and outcome, preferably on an interpretable scale	Figure 3 B-G;<br>Table 2. MR estimates of markers.
		b)	Report MR estimates of the relationship between exposure and outcome, and the measures of uncertainty from the MR analysis, on an interpretable scale, such as odds ratio or relative risk per SD difference	Supplementary Table S2
		c)	If relevant, consider translating estimates of relative risk into absolute risk for a meaningful time period	NA
		d)	Consider plots to visualize results (e.g. forest plot, scatterplot of associations between genetic variants and outcome versus between genetic variants and exposure)	Figure 5A-I;<br>Figure 3A-J;<br>Supplementary Figure S8, 11.
12	**Assessment of assumptions**			
		a)	Report the assessment of the validity of the assumptions	Supplementary Table S2-4.
		b)	Report any additional statistics (e.g., assessments of heterogeneity across genetic variants, such as I^2, Q statistic or E-value)	Table 3. Marker MR results and heterogeneity and horizontal pleiotropy tests.
13	**Sensitivity analyses and**			

	**additional analyses**		
	a)	Report any sensitivity analyses to assess the robustness of the main results to violations of the assumptions	MR-Egger intercepts, MR-PRESSO and Leave-one-out MR.
	b)	Report results from other sensitivity analyses or additional analyses	Table 3. Marker MR results and heterogeneity and horizontal pleiotropy tests;<br>Figure 7. The causality of $CD4^+$ T_{EM} markers and PTB/COVID-19 co-infection based on MR analysis.
	c)	Report any assessment of direction of causal relationship (e.g., bidirectional MR)	Reverse MR analysis (Figure 3K, Figure 5I)
	d)	When relevant, report and compare with estimates from non-MR analyses	Paragraph 3 in Discussion
	e)	Consider additional plots to visualize results (e.g., leave-one-out analyses)	Figure 5A-I;<br>Figure 3A-J;<br>Supplementary Figure S8, 11.
	**DISCUSSION**		
14	**Key results**	Summarize key results with reference to study objectives	Paragraph 1 in Discussion
15	**Limitations**	Discuss limitations of the study, taking into account the validity of the IV assumptions, other sources of potential bias, and imprecision. Discuss both direction and magnitude of any potential bias and any efforts to address them	The last paragraph of Discussion
16	**Interpretation**		
	a)	Meaning: Give a cautious overall interpretation of results in the context of their limitations and in comparison with other studies	Paragraphs 3, 4 in Discussion
	b)	Mechanism: Discuss underlying biological mechanisms that could drive a potential causal relationship between the investigated exposure and the outcome, and whether the gene-environment equivalence assumption is reasonable. Use causal language carefully, clarifying that IV estimates may provide causal effects only under certain assumptions	Paragraphs 3, 4 in Discussion
	c)	Clinical relevance: Discuss whether the results have clinical or public policy relevance, and to what extent they inform effect sizes of possible interventions	Paragraphs 5 in Discussion
17	**Generalizability**	Discuss the generalizability of the study results (a) to other populations, (b) across other exposure periods/timings, and (c) across other levels of exposure	Paragraph 2, 3, 4 in Discussion
	**OTHER INFORMATION**		

18	**Funding**	Describe sources of funding and the role of funders in the present study and, if applicable, sources of funding for the databases and original study or studies on which the present study is based	The Funding section
19	**Data and data sharing**	Provide the data used to perform all analyses or report where and how the data can be accessed, and reference these sources in the article. Provide the statistical code needed to reproduce the results in the article, or report whether the code is publicly accessible and if so, where	The Data Availability Statement section
20	**Conflicts of Interest**	All authors should declare all potential conflicts of interest	The Conflict of Interest section

This checklist is copyrighted by the Equator Network under the Creative Commons Attribution 3.0 Unported (CC BY 3.0) license.

1. Skrivankova VW, Richmond RC, Woolf BAR, Yarmolinsky J, Davies NM, Swanson SA, et al. Strengthening the Reporting of Observational Studies in Epidemiology using Mendelian Randomization (STROBE-MR) Statement. JAMA. 2021;under review.

2. Skrivankova VW, Richmond RC, Woolf BAR, Davies NM, Swanson SA, VanderWeele TJ, et al. Strengthening the Reporting of Observational Studies in Epidemiology using Mendelian Randomisation (STROBE-MR): Explanation and Elaboration. BMJ. 2021;375:n2233.
