# Supplementary material for: CD4+ Effective Memory T Cell Markers GBP2 and LAG3 Are Risk Factors for PTB and COVID-19 Infection: A Study Integrating Single-Cell Expression Quantitative Trait Locus and Mendelian Randomization Analyses
Source: Int J Mol Sci. 2024 Sep 16;25(18):9971. doi: 10.3390/ijms25189971 (PMC11432203; doi:10.3390/ijms25189971)
Supplement: Supplementary file 1 [file ijms-25-09971-s001.zip › Supplementary material/Supplementary Figures S1-S12.docx]

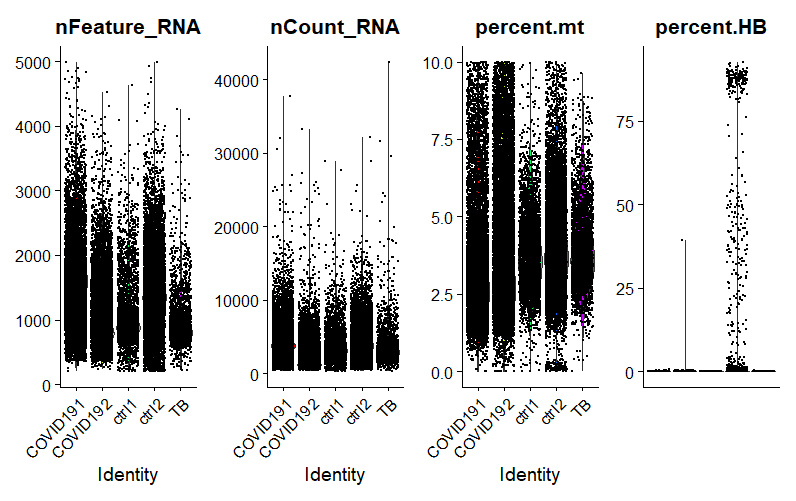


**Fig. S1. Quality control analysis of single-cell sequencing RNA.** The quantity of characteristic RNA (nFeature_RNA). the number of recognised RNA (nCount_RNA), the proportion of mtRNA (percent. mt), and the ratio of red blood cell gene expression (percent. HB) in each cell of all PBMCs.


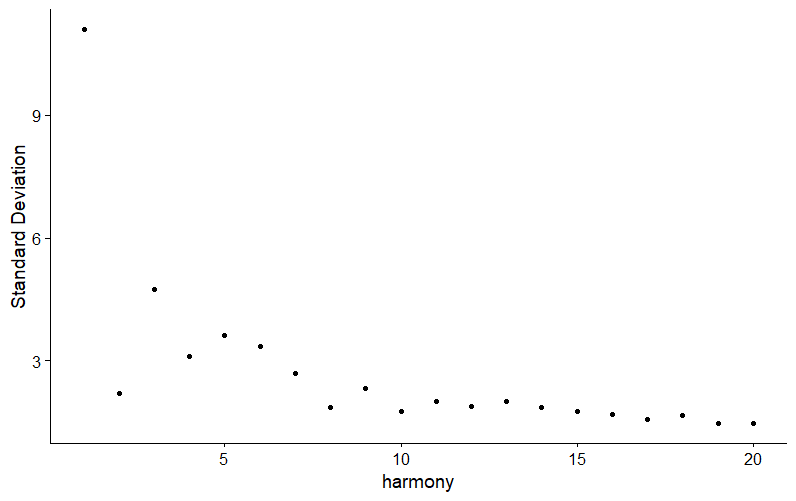


**Fig. S2. The ElbowPlot of principal components** **before all PBMC clustering.** ElbowPlot was used to identify the significantly available dimensions of data sets with estimated P value and Elbow. The horizontal coordinate represents the number of PCs, and the vertical coordinate represents the standard deviation, which shows that the standard deviation of the principal component (PC) of the two samples did not change significantly after harmony=10, so the most obvious 10 principal components were selected for the subsequent cluster analysis.


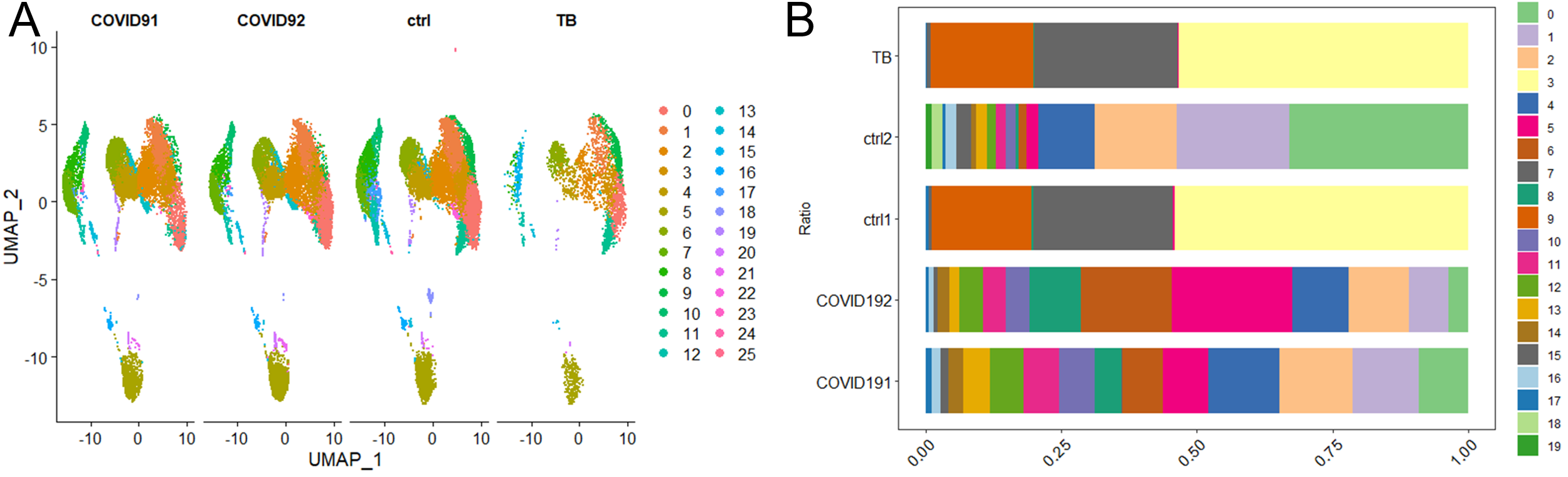


**Fig. S3.** The results of the PC analysis were clustered to obtain 25 cell clusters and the distribution of the cells was shown in two dimensions by reducing the dimensionality of the cells using UMAP (A) and histogram (B).


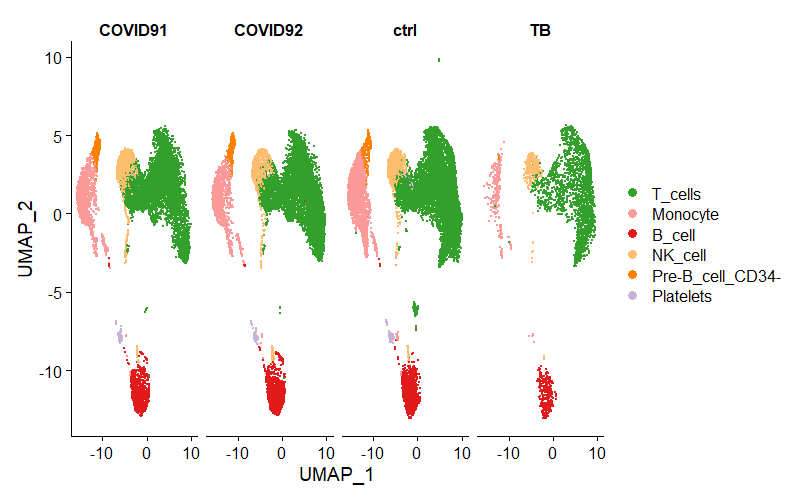


**Fig. S4. Spatial distribution of UMAP after cell clusters** **naming using the “Seurat” R package.**


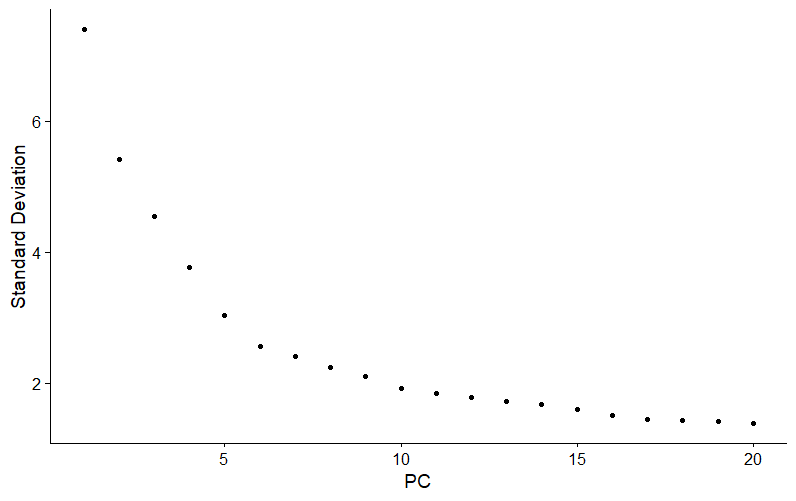


**Fig. S5. The ElbowPlot of principal components** **after all PBMC clustering.** ElbowPlot was used to identify the significantly available dimensions of data sets with estimated P value and Elbow. The horizontal coordinate represents the number of PCs, and the vertical coordinate represents the standard deviation, which shows that the standard deviation of the principal component (PC) of the two samples did not change significantly after harmony=10, so the most obvious 10 principal components were selected for the subsequent cluster analysis.


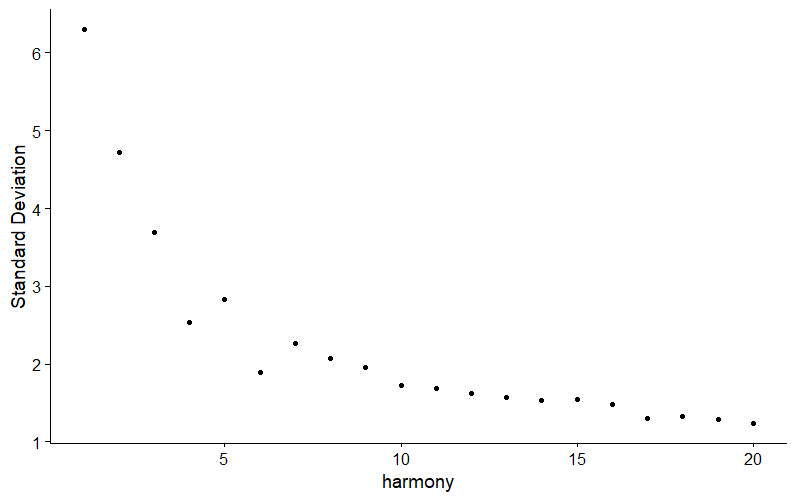


**Fig. S6. The ElbowPlot of principal components** **before T cells clustering.** ElbowPlot was used to identify the significantly available dimensions of data sets with estimated P value and Elbow. The horizontal coordinate represents the number of PCs, and the vertical coordinate represents the standard deviation, which shows that the standard deviation of the principal component (PC) of the two samples did not change significantly after harmony=10, so the most obvious 10 principal components were selected for the subsequent cluster analysis.


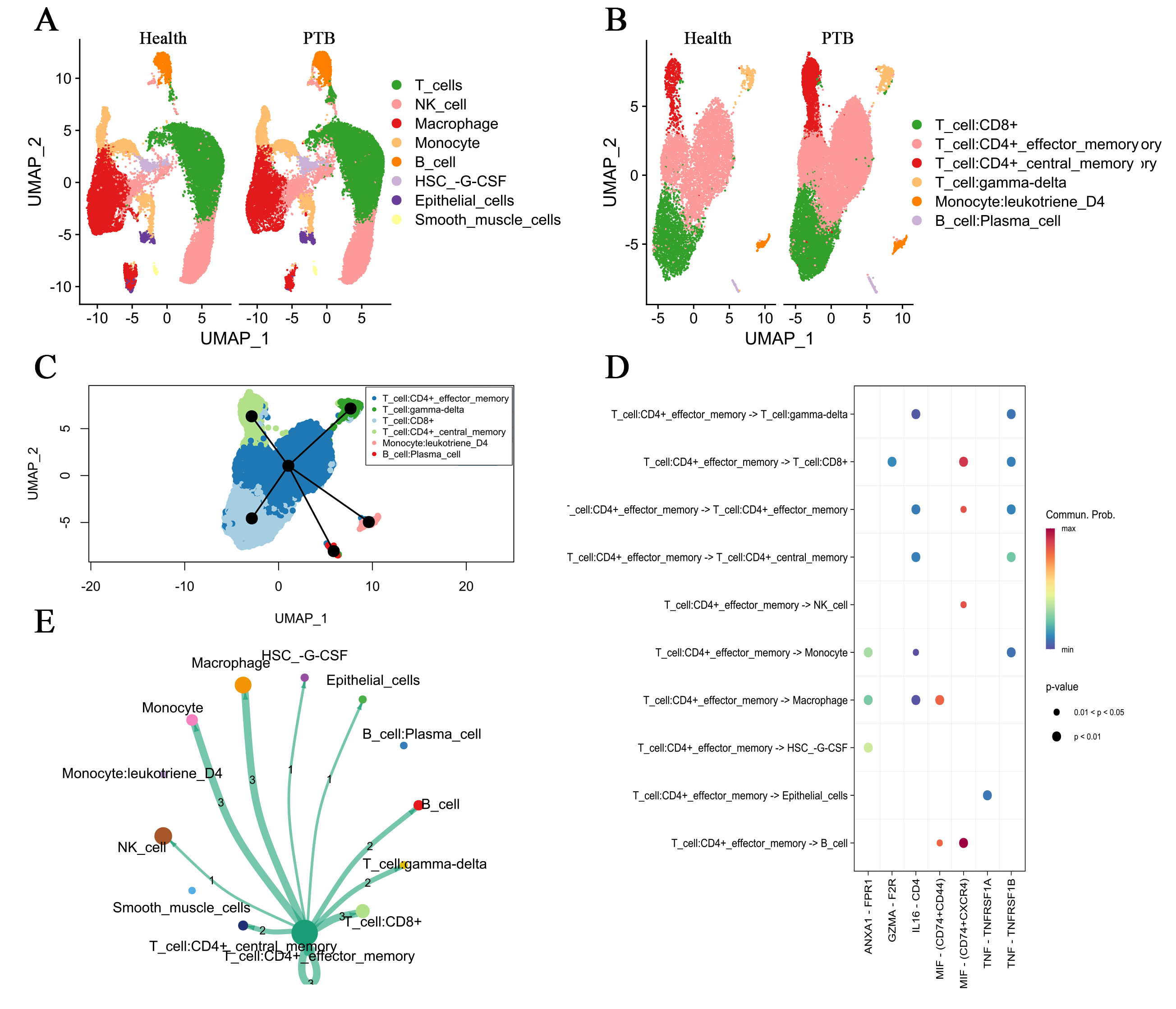


**Figure S7.** Single-cell transcriptional landscape of lung tissue from PTB patients. patients met the following Inclusion criteria: (1) regular treatment of sputum continuous positive or repeated positive localized lesions; (2) the presence of tuberculosis cavity, destruction of lung and other that can cause sputum negative tuberculosis recurrence; (3) tuberculosis-led bronchitis, tube pleural fistula, empyema, hemoptysis or malignancy; and, (4) no cardiovascular, cerebrovascular, hypertension, diabetes and other surgical contraindications, as previously described. Sputum-positive patients were excluded to continue treatment as per guideline recommendation. Exclusion criteria were HIV-positive, malignancy, recent immunosuppressive medication, pregnancy, and anemia. (**A**) The spatial distribution of each cell subset is named and visualized using UMAP. (**B**) Visualization of T cell subsets by UMAP diagram. (**C**) Single-cell gene expression in a seven-dimensional PCA plot with branching lineage trajectories determined with the "Slingshot" package of R. (**D, E**) Network of cell-cell communication that shows the quantity of receptor-ligand pairings between CD4^+^ T_EM_ cells and other cell subsets associated with PTB.


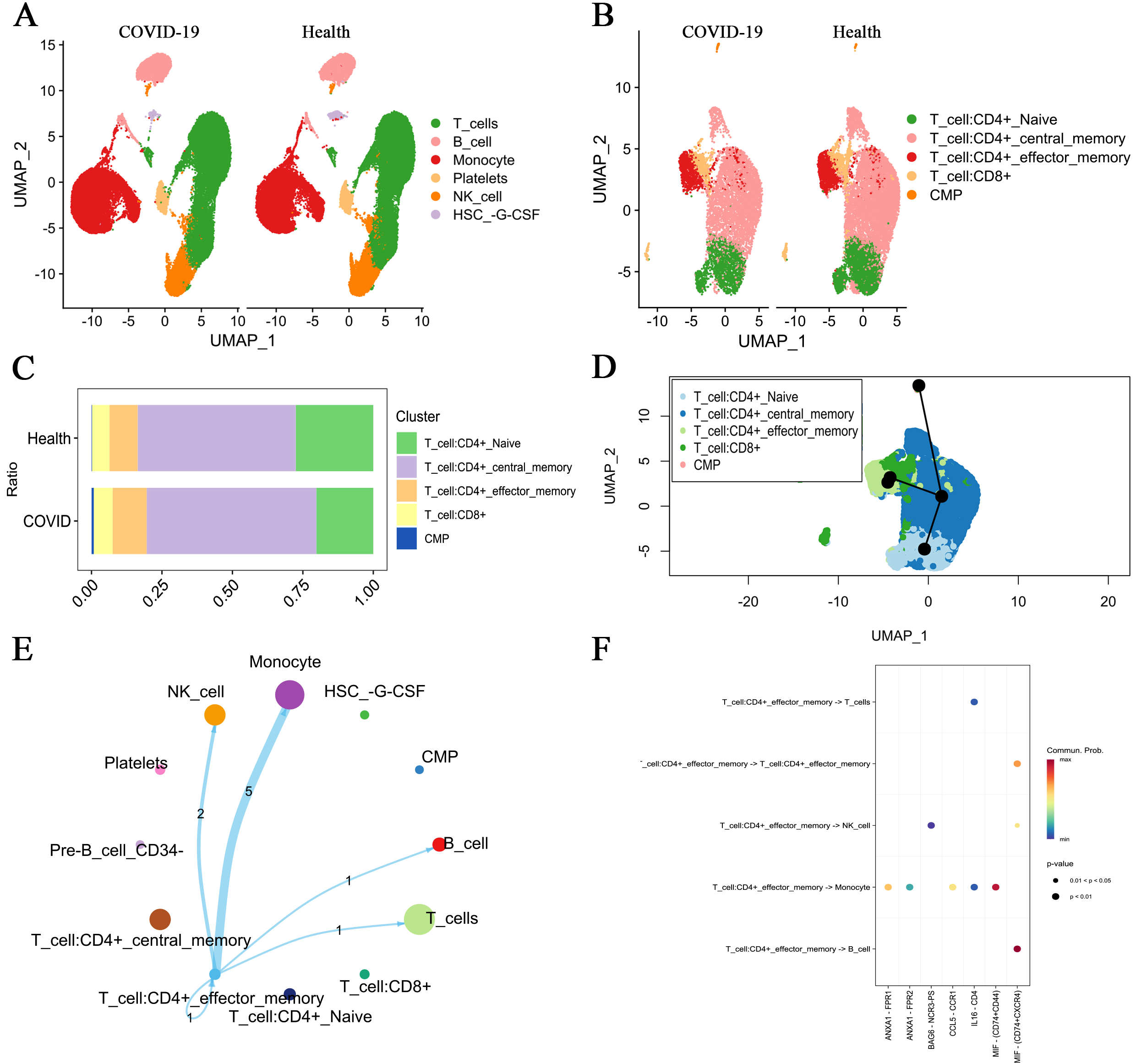


**Figure S8.** Single-cell transcriptional landscape of PBMC from COVID-19 patients. Patients included those with critical COVID-19 defined by the requirement for admission to the intensive care unit. (**A**) The spatial distribution of each cell subset is named and visualized using UMAP. (**B**) Visualization of T cell subsets by UMAP diagram and histogram (**C**). (**D**) Single-cell gene expression in a seven-dimensional PCA plot with branching lineage trajectories determined with the "Slingshot" package of R. (**E, F**) Network of cell-cell communication that shows the quantity of receptor-ligand pairings between CD4^+^ T_EM_ cells and other cell subsets associated with COVID-19.


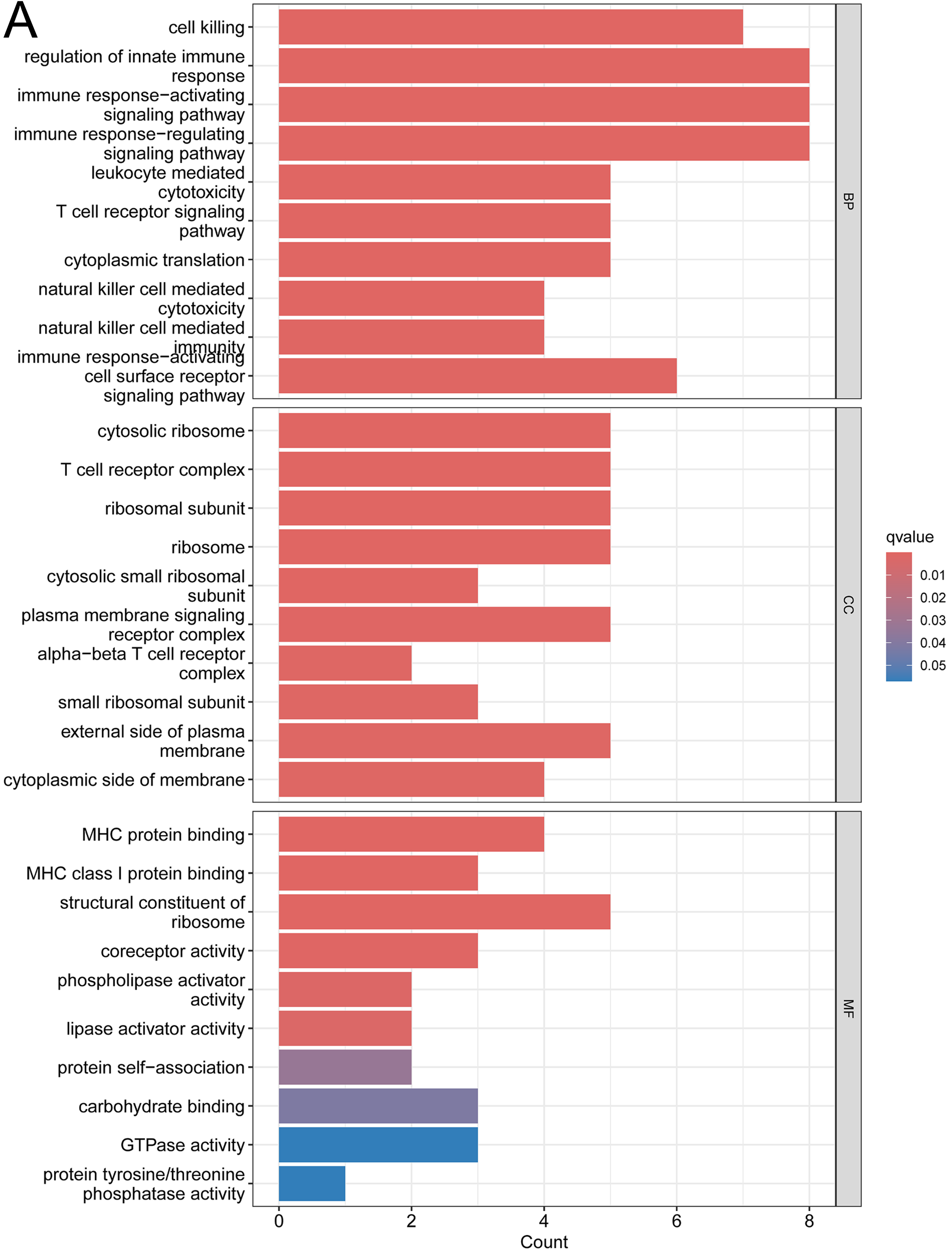

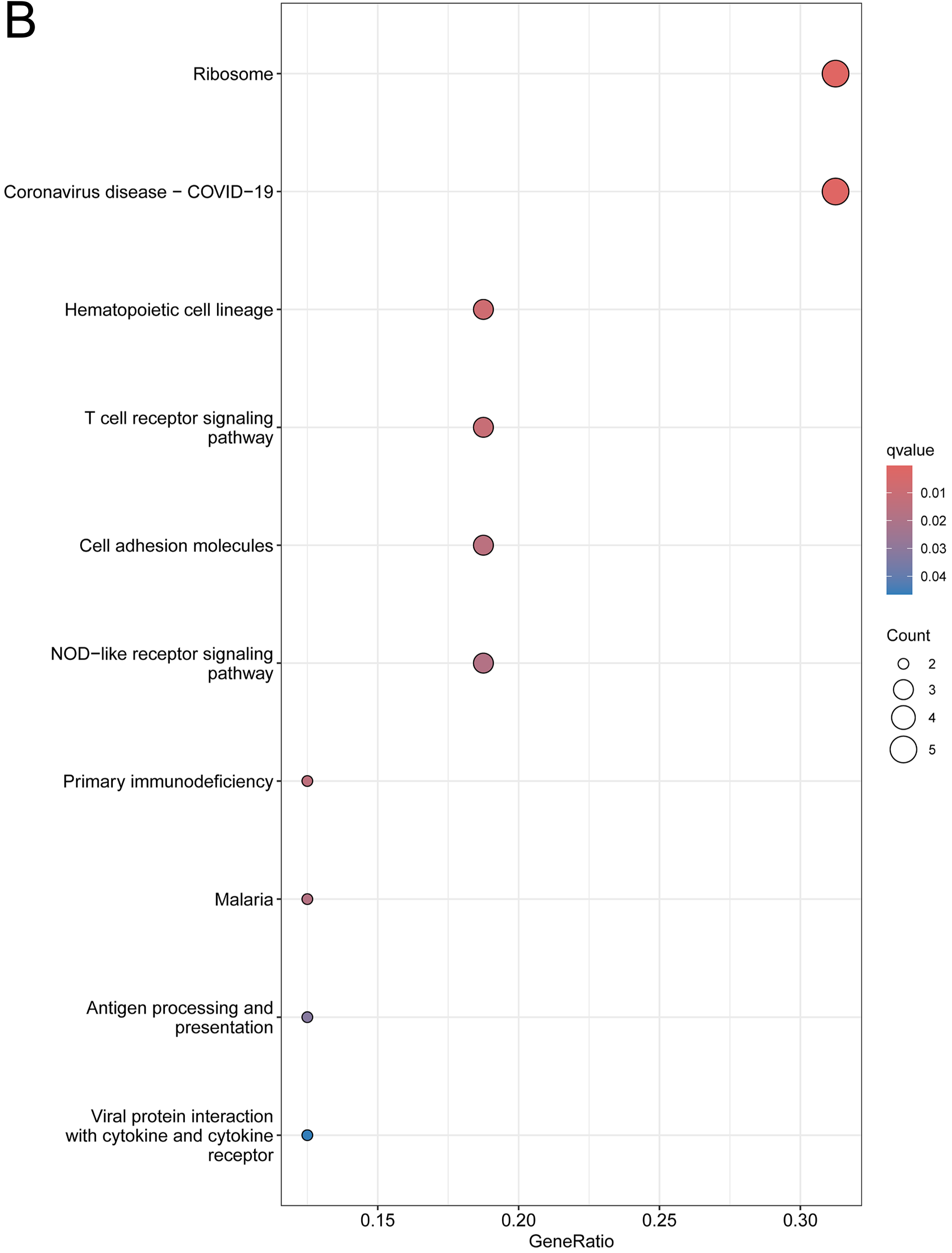


**Fig. S9. GO & KEGG analysis of these 30 markers.**

**
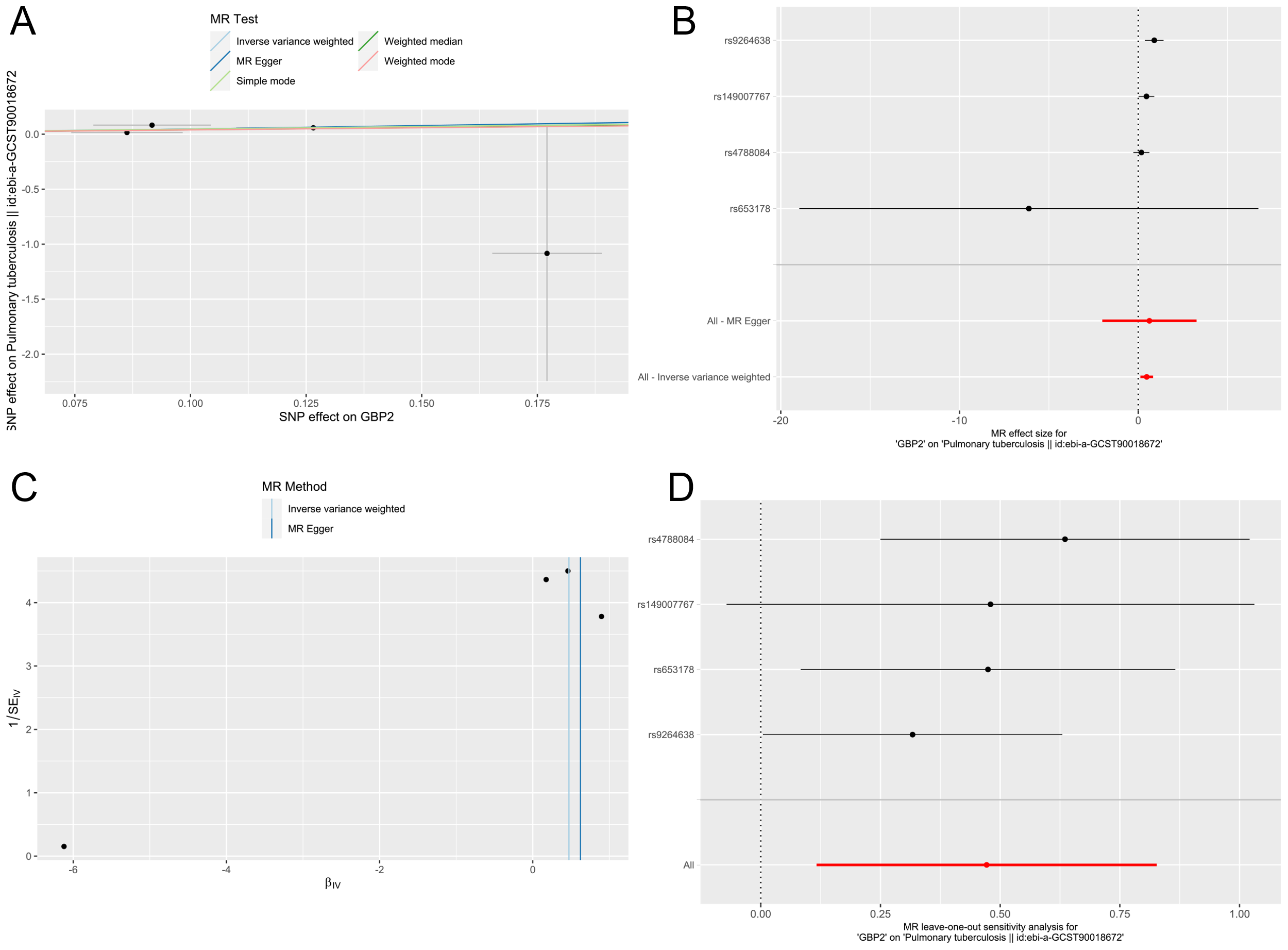
**

**Fig. S10.** **(A)** Scatter plots of 5 MR models. Each point represents an IV, the line on each point represents a 95% confidence interval (CI), the abscissa is the effect of SNP on exposure, and the ordinate is the effect of SNP on outcome. **(B)** Forest plot of MR analysis results for single SNP estimation of GBP2. **(C)** Funnel plot of three SNPs on MR analysis. **(D)** MR sensitivity results of GBP2 after removing SNP by the leave-one-out method.


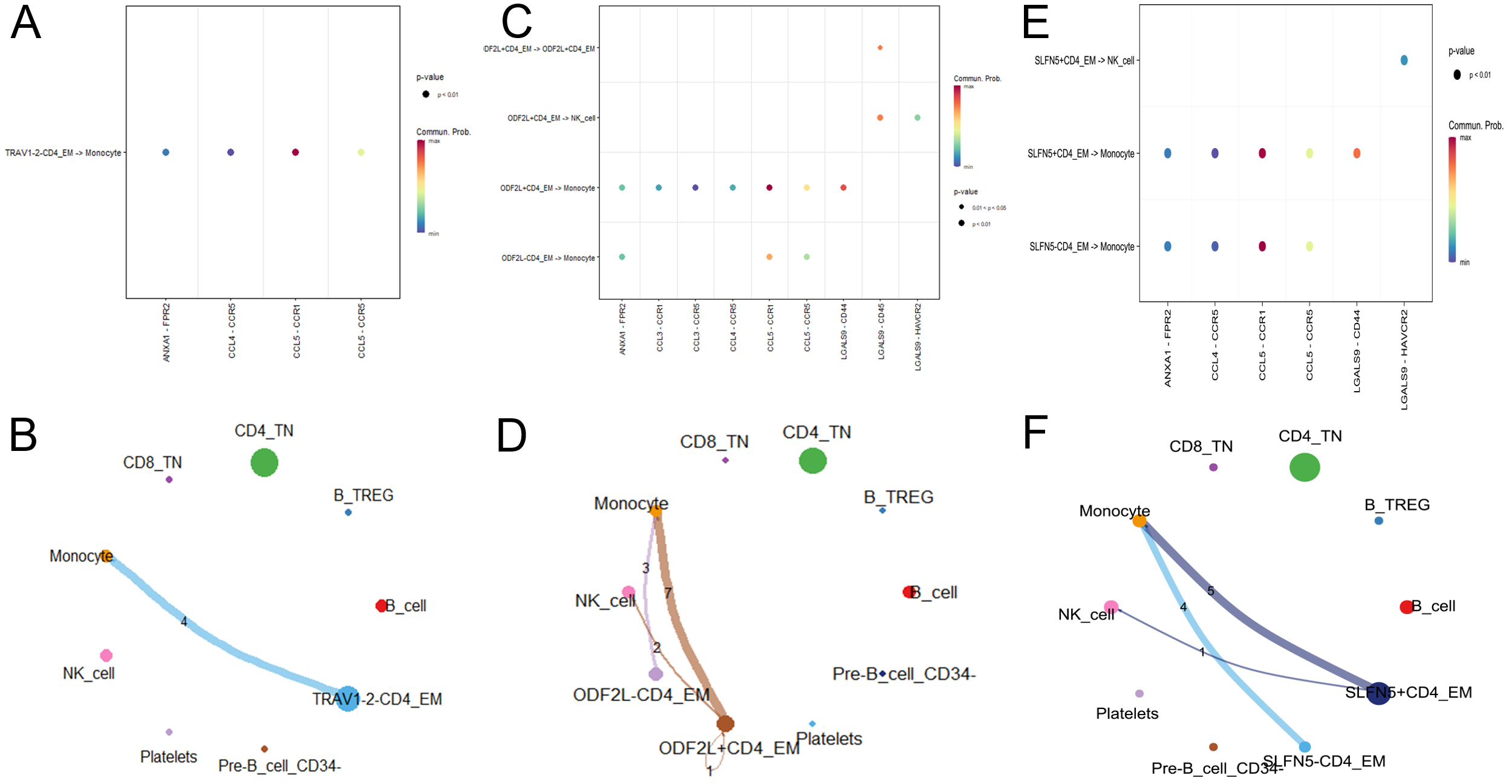


**Fig. S11.** The cell-cell communication network displays the number of receptor-ligand pairs between markers-positive and negative CD4^+^ T_EM_ cells and other T-cell subsets. The thickness of these lines represents the number of pairs. TRAV1-2 **(A, B)**; ODF2L **(C, D)**; SLFN5 **(E, F)**


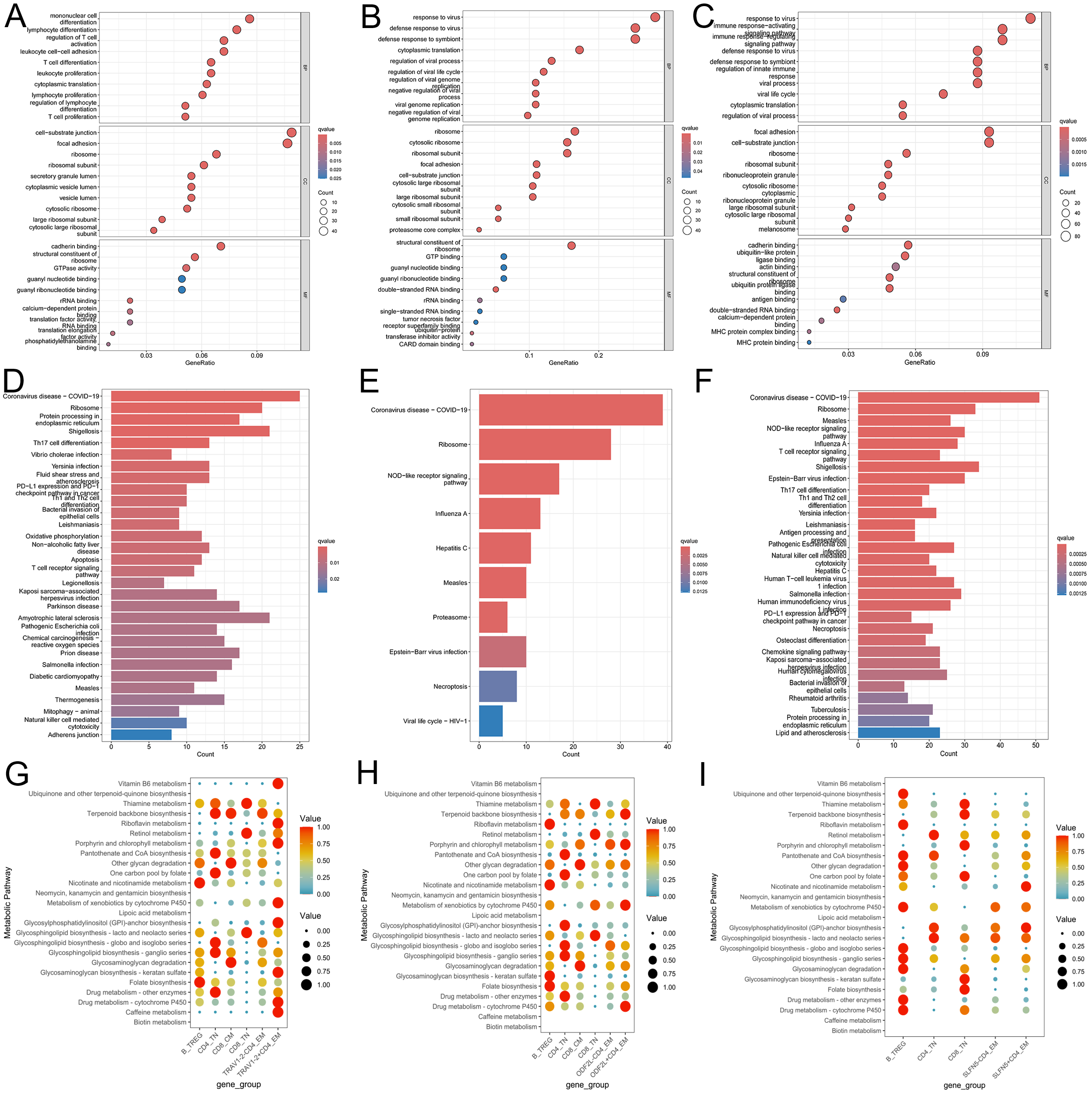


**Fig. S12.** (A-C) GO analysis of markers-positive and negative CD4^+^ T_EM_ cells. TRAV1-2, ODF2L, and SLFN5, respectively. (D-F) KEGG analysis of markers-positive and negative CD4^+^ T_EM_ cells. TRAV1-2, ODF2L, and SLFN5, respectively. (G-I) Bubble diagram of metabolic pathways enrichment based on the differential metabolites among markers-positive and negative CD4^+^ T_EM_ cells. One bubble represents one metabolic pathway. The numbers of involved metabolites and the P-value were listed on the right side. TRAV1-2, ODF2L, and SLFN5, respectively.


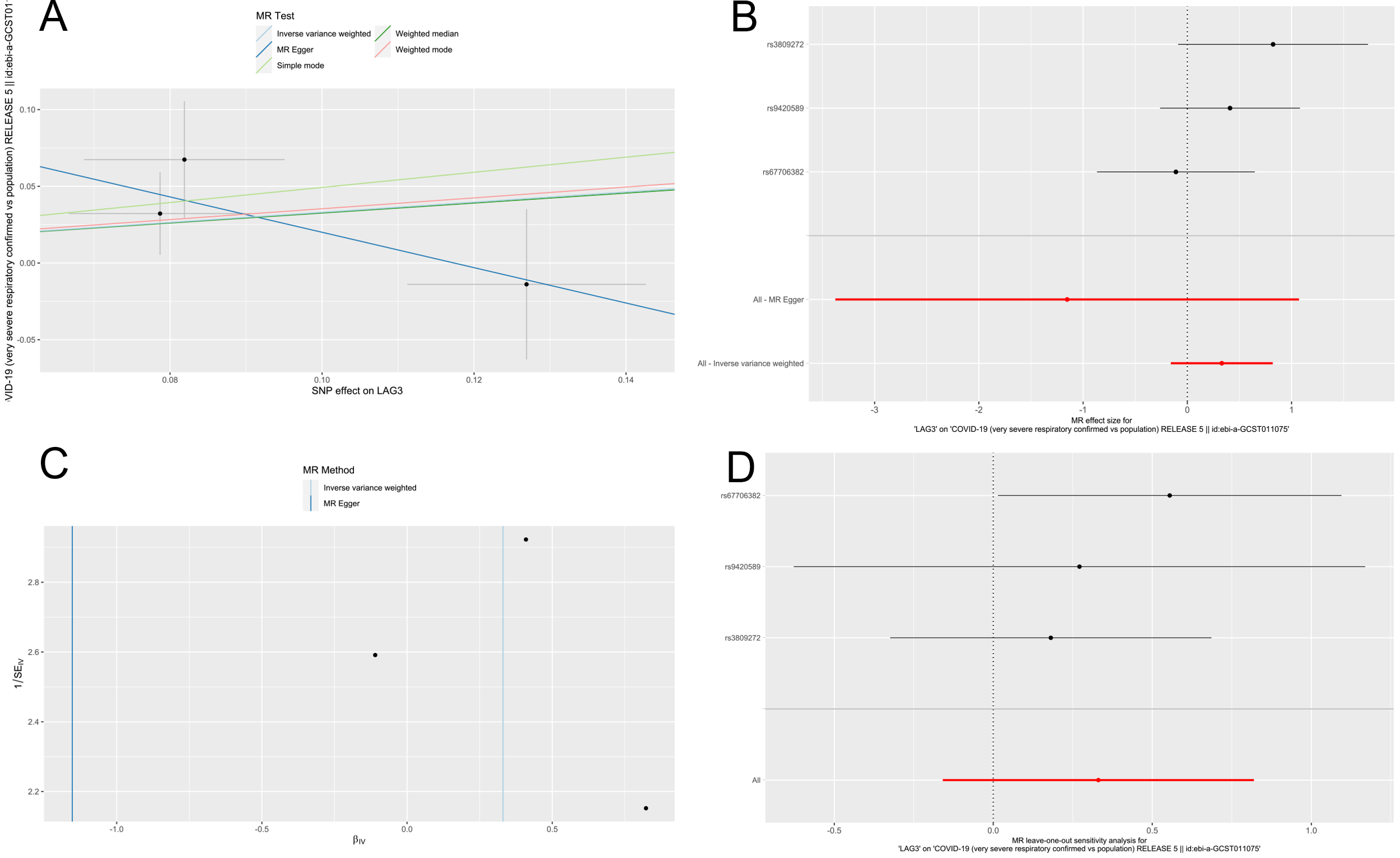


**Fig. S13.** **(A)** Scatter plots of 5 MR models. Each point represents an IV, the line on each point represents a 95% confidence interval (CI), the abscissa is the effect of SNP on exposure, and the ordinate is the effect of SNP on outcome. **(B)** Forest plot of MR analysis results for single SNP estimation of LAG3. **(C)** Funnel plot of three SNPs on MR analysis. **(D)** MR sensitivity results of LAG3 after removing SNP by the leave-one-out method.


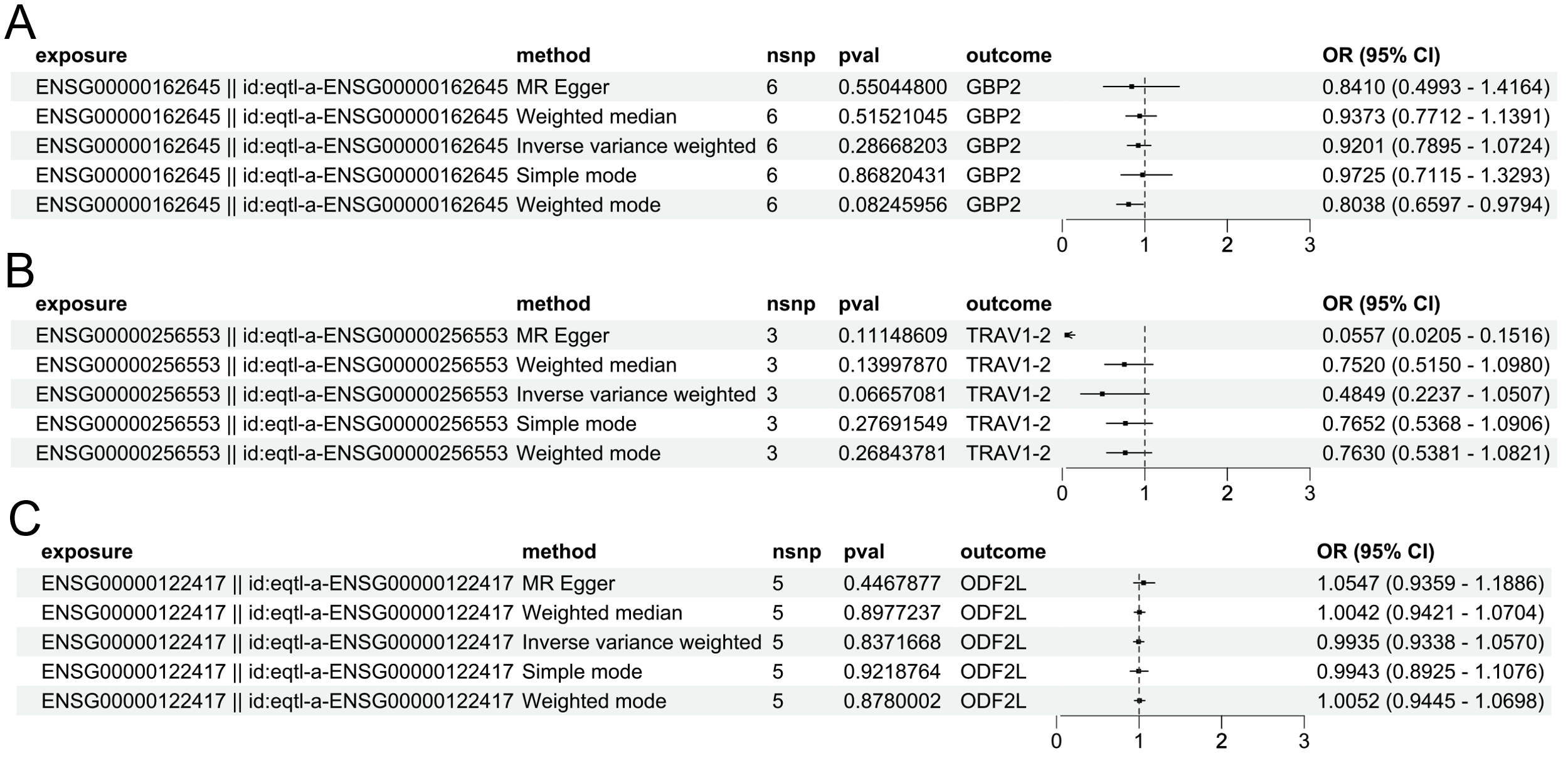


**Fig. S14.** MR analysis of GBP2, TRAV1-2, and ODF2L (A, B, and C respectively) and COVID-19.
